# Decreased expression of mitochondrial miR-5787 contributes to chemoresistance by reprogramming glucose metabolism and inhibiting MT-CO3 translation

**DOI:** 10.7150/thno.37556

**Published:** 2019-08-12

**Authors:** Weixiong Chen, Peng Wang, Yingjuan Lu, Tingting Jin, Xinyuan Lei, Mo Liu, Peilin Zhuang, Junkun Liao, Zhaoyu Lin, Bowen Li, Yu Peng, Guokai Pan, Xiaobin Lv, Hanqing Zhang, Zhanpeng Ou, Shule Xie, Xinyu Lin, Sheng Sun, Soldano Ferrone, Bakhos A. Tannous, Yi Ruan, Jinsong Li, Song Fan

**Affiliations:** 1 Guangdong Provincial Key Laboratory of Malignant Tumor Epigenetics and Gene Regulation of Sun Yat-Sen Memorial Hospital, Guangzhou 510120, China; 2 Department of Oral and Maxillofacial Surgery, Sun Yat-Sen Memorial Hospital of Sun Yat-Sen University, Guangzhou 510120, China; 3 Division of Nephrology, Nanfang Hospital, Southern Medical University, State Key Laboratory of Organ Failure Research, National Clinical Research Center of Kidney Disease, Guangzhou 510515, China.; 4 State University of New York at Stony Brook, Stony Brook, NY, 11794, USA; 5 Markey Cancer Center, the University of Kentucky, College of Medicine, Lexington, Kentucky 40506, USA; 6 Nanchang Key Laboratory of Cancer Pathogenesis and Translational Research, Center Laboratory, the Third Affiliated Hospital, Nanchang University, Nanchang 330047, China; 7 Massachusetts General Hospital Cancer Center, Harvard Medical School, Boston, MA02114, USA; 8 Department of Surgery, Massachusetts General Hospital, Harvard Medical School, 55 Fruit Street, Boston, MA 02114, USA; 9 Experimental Therapeutics and Molecular Imaging Lab, Department of Neurology, Massachusetts General Hospital and Harvard Medical School, Boston, MA 02129, USA

**Keywords:** mitochondrial miR-5787, oxidative phosphorylation, aerobic glycolysis, cisplatin chemotherapy resistance, MT-CO3, tongue squamous cell carcinoma

## Abstract

MicroRNAs (miRNAs) have been recently found in the mitochondria, and were named “mitomiRs”, but their function has remained elusive. Here, we aimed to assess the presence and function(s) of mitomiRs in tongue squamous cell carcinoma (TSCC).

**Methods:** miRNA microarray was performed in paired TSCC cell lines, Cal27 and its chemoresistant counterpart, Cal27-re. Decreased expression of mitomiRs in chemoresistant cells was characterized. The functions of mitomiRs were investigated by a series of *in vitro* and *in vivo* experiments.

**Results:** Differential microarray analysis identified downregulation of mitomiR-5787 in Cal27-re cells. We knocked down mitomiR-5787 in parental cells and upregulated its expression in cisplatin-resistant cells. The sensitivity of TSCC cells to cisplatin was regulated by miR-5787. The glucose metabolism assay suggested that reduced expression of miR-5787 changed the balance of glucose metabolism by shifting it from oxidative phosphorylation to aerobic glycolysis. Xenograft experiments in BALB/c-nu mice further verified the *in vitro* results. Reduced expression of miR-5787 contributes to chemoresistance in TSCC cells by inhibiting the translation of mitochondrial cytochrome c oxidase subunit 3 (MT-CO3). The prognostic analysis of 126 TSCC patients showed that the patients with low expression of miR-5787 and/or MT-CO3 had poor cisplatin sensitivity and prognosis.

**Conclusions:** Mitochondrial miR-5787 could regulate cisplatin resistance of TSCC cells and affect oxidative phosphorylation and aerobic glycolysis. Downregulation of miR-5787 inhibited the translation of MT-CO3 to regulate cisplatin resistance of TSCC. Mitochondrial miR-5787 and MT-CO3 can be used as predictive biomarkers or therapeutic targets for cisplatin chemotherapy resistance.

## Introduction

Cancer cells exhibit altered glucose metabolism compared to normal cells with a preference for glycolysis. This hallmark of cancer cells was first described in the 1920s by Otto Warburg and is known as the “Warburg effect” 1. Based upon this distinctive characteristic, positron emission tomography-computed tomography (PET-CT) can be used for the analysis of cancer cells. It is well recognized that in cancer cells, oxidative phosphorylation (OXPHOS), the main metabolic pattern of the normal cells is decreased. Even under high oxygen tension, cancer cells favor aerobic glycolysis and generate more lactate compared to normal cells allowing them to meet the requirements of rapid growth 2. OXPHOS is exclusively regulated by mitochondria and the enhancement of this mitochondrial function may endow cancer cells with malignant characteristics. The metabolism of cancer cells appears to play a crucial role in the evolution and progression of tumors. Some cancer cells depend on OXPHOS for survival which is also responsible for tumor relapse 3. It has been reported that both normal and leukemic stem cells rely on mitochondrial respiration to generate more energy 4. Mitochondrion not only is a cellular powerhouse but is also implicated in many other essential cellular processes, such as growth, differentiation, apoptosis, and cell death 5.

MicroRNAs (miRNAs), with a length of 19-25 nucleotides, are a class of endogenous small noncoding RNAs that exert their functions through posttranscriptional regulation of gene expression. In mammalian cells, miRNAs can bind to mRNA transcripts with complementary sequences, usually in the 3' untranslated region (UTR), inhibiting mRNA translation and/or inducing mRNA degradation 6. Multiple studies have revealed the critical role of miRNAs in the development and progression of tumors. Some of these miRNAs influence cancer progression by regulating tumor metabolism. miRNAs can reprogram glucose metabolism to affect metastasis 7, proliferation 8, 9, cell cycle distribution 9, and apoptosis 9.

Recently, miRNAs have also been detected in mitochondria and considered to have an association with metabolism and other cellular functions. Nucleus-encoded miRNAs were identified in highly purified liver-derived mitochondria from adult rats 10, and some unique miRNAs were specifically enriched within mouse liver mitochondria 11; the functions of these miRNAs, however, remained elusive. Recently, a study revealed that miR-181c translocated into the mitochondria, where it inhibited the translation of mt-COX1, causing electron transport chain complex IV remodeling and increasing production of reactive oxygen species (ROS) in cardiac myocytes 12. In contrast to the usual mechanism by which miRNAs function in the cytoplasm, some reports indicated that mitochondrial miRNAs (mitomiRs) can enhance mitochondrial translation, regulating cell differentiation 13 or blood pressure 14.

In this study, we characterized, for the first time, mitomiRs in tongue squamous cell carcinoma (TSCC) cells. Among these mitomiRs, miR-5787 was downregulated in cisplatin-resistant TSCC cells, resulting in the downregulation of mitochondrial cytochrome c oxidase subunit 3 (MT-CO3), a metabolic shift from OXPHOS to glycolysis, and chemotherapy resistance in TSCC cells. Our findings not only demonstrate the critical role of mitomiRs in the metabolic changes that occur during chemotherapy in cancer cells but also identify the involvement of mitomiRs in chemotherapy resistance of cancer cells.

## Methods

### Cell culture

The TSCC cell lines Cal27 and Scc25 were purchased from the American Type Culture Collection. The stable cisplatin-resistant lines Cal27-re and Scc25-re were established by treating Cal27 or Scc25 with cisplatin (Sigma, St. Louis, MO, USA) at concentrations of 10^-7^ M to 10^-5^ M. Cal27 and Cal27-re cells were cultivated in Dulbecco's modified Eagle's medium (Gibco, Rockville, MD, USA) supplemented with 10% fetal bovine serum (Biological Industries). Scc25 and Scc25-re cells were cultivated in Dulbecco's modified Eagle's medium-F12 (Gibco) supplemented with 10% fetal bovine serum (Biological Industries).

### Quantification of mtDNA and miR-5787

To quantify the copy number of miR-5787 per nucleus or mitochondrial genome, we generated a standard curve by qRT-PCR using a series dilution of synthetic miR-5787 from Ribobio (Guangzhou, China) and then performed quantitative analysis of miR-5787 in whole-cell lysate or purified mitochondria.

### Apoptosis assay

After the indicated treatments, the cells were exposed to the IC50 of cisplatin (Sigma) for 24 h for the apoptosis assay. Transferase dUTP nick end labeling (TUNEL) assay was performed using a kit from Roche (Cat. No. 11684795910). The detection procedures were performed following the kit instructions. Sections were examined with an ImagerZ1 microscope (Zeiss, Jena, Germany). An investigator who was blinded to the treatment quantified 20 random fields in the samples.

### Mitochondrial respiratory chain complex activity

The activities of mitochondrial respiratory chain complexes I, II, III, IV, and V were measured according to the instructions provided with the appropriate kits (GMS50007, GMS50008, GMS50009, GMS50010, and GMS50083) from GENMED (Shanghai, China). Briefly, mitochondria were collected from Cal27, Cal27-re, Scc25, and Scc25-re cells, and the reagents from 5 GENMED kits were applied. Finally, optical density (OD) values were measured and subjected to analysis.

### Glucose uptake assay

The glucose consumption rate was assessed with an assay kit (Nanjing Jiancheng Bioengineering Institute, F006). Briefly, the culture medium was collected after the indicated treatment for 24 h in parental and chemoresistant TSCC cells. The glucose consumption rate was calculated and normalized to that of fresh culture medium.

### ATP assay

ATP was measured by an ATP Assay Kit (Beyotime Biotechnology, S0026). Briefly, protein extracts were suspended in standard reaction buffer containing luciferin and luciferase according to the manufacturer's instructions, and luminescence was read at 560 nm.

### Lactate production assay

Lactate production was measured with a lactic acid assay kit (Nanjing Jiancheng Bioengineering Institute, A019-2). Briefly, the chemoresistant cells were cultured in 6-well plates and treated with miR-5787 mimics for 24 h, the culture medium was collected, and the lactate concentration was measured according to the manufacturer's protocols. The lactate production in parental cells transfected with the miR-5787 sponge was also compared to that in the mock and miR sponge-NC in parental cells.

### pH measurement

The intracellular pH (pHin) of cells was assessed by flow cytometry using the pH-sensitive fluorescent probe BCECF-AM (Beyotime Biotechnology, S1006). The cell suspension in serum-free culture medium was washed and labeled with BCECF-AM. The labeled cells were analyzed at an excitation wavelength of 488 nm, and the ratio of the fluorescence at 530 nm to that at 640 nm was plotted versus pHin. To obtain the calibration curve, a linear regression within the pHin range 6.2-7.4 was obtained. The extracellular pH (pHex) of the cells was measured by monitoring culture medium pH changes with a pH meter.

### Tumor xenografts

The animal experiments were approved by Sun Yat-sen University laboratory animal care and use committee. Male BALB/c-nu mice (4-6 weeks old) were randomly divided into 6 groups (n=6) and applied to evaluate the effects of miR-5787 on TSCC cells *in vivo*. In total, 5 × 10^6^ cells resuspended in 150 μL of PBS were injected subcutaneously into the flanks of the nude mice. One week after implantation, when the tumor became palpable with a diameter of ~2 mm, cisplatin (5 mg/kg) was administered via intraperitoneal injections every three days from day 8 to 32. On day 35, the primary tumors were carefully removed, imaged, and analyzed by immunohistochemistry (IHC), *in situ* hybridization (ISH), Western blots, and qRT-PCR.

### Patients and tissue samples

Specimens of locally advanced TSCC patients (n = 126) were obtained between Jan 1, 2004, and Dec 31, 2010. The patients were treated with cisplatin prior to surgery. According to the 'Response Evaluation Criteria in Solid Tumors' of the World Health Organization, TSCC with progressive disease or a stable disease response were designated cisplatin- resistant or cisplatin-insensitive TSCC, whereas TSCC that showed a partial or complete response were determined to be cisplatin-sensitive TSCC. The tissues were obtained from the respective pathology departments, and histological diagnosis and scoring of all cases were performed by two independent pathologists. Survival time was calculated from the date of surgery to the date of death or the last follow-up. The date of death was obtained from patient records or through follow-up telephone calls. This study was approved by the institutional ethical review boards of our hospital, and written informed consent was obtained from all patients.

### Statistics

All statistical analyses were performed using SPSS 19.0 (SPSS, Chicago, IL, USA). Student's t-test and the Chi-square test were used to analyze the relationships between miR-5787 or MT-CO3 expression and clinicopathological characteristics. To measure the associations between pairs of variables, Spearman's rank order correlations were performed. Kaplan-Meier survival curves were plotted, and a log-rank test was performed. All cell culture experiments were performed with at least three independent experiments. The data were expressed as the mean ± the standard error of the mean (SEM). *P*<0.05 was considered a significant difference statistically.

Additional Materials and Methods are detailed in Supplementary data.

## Results

### Screening for functional mitomiRs in TSCC cells

**Figure 1:**
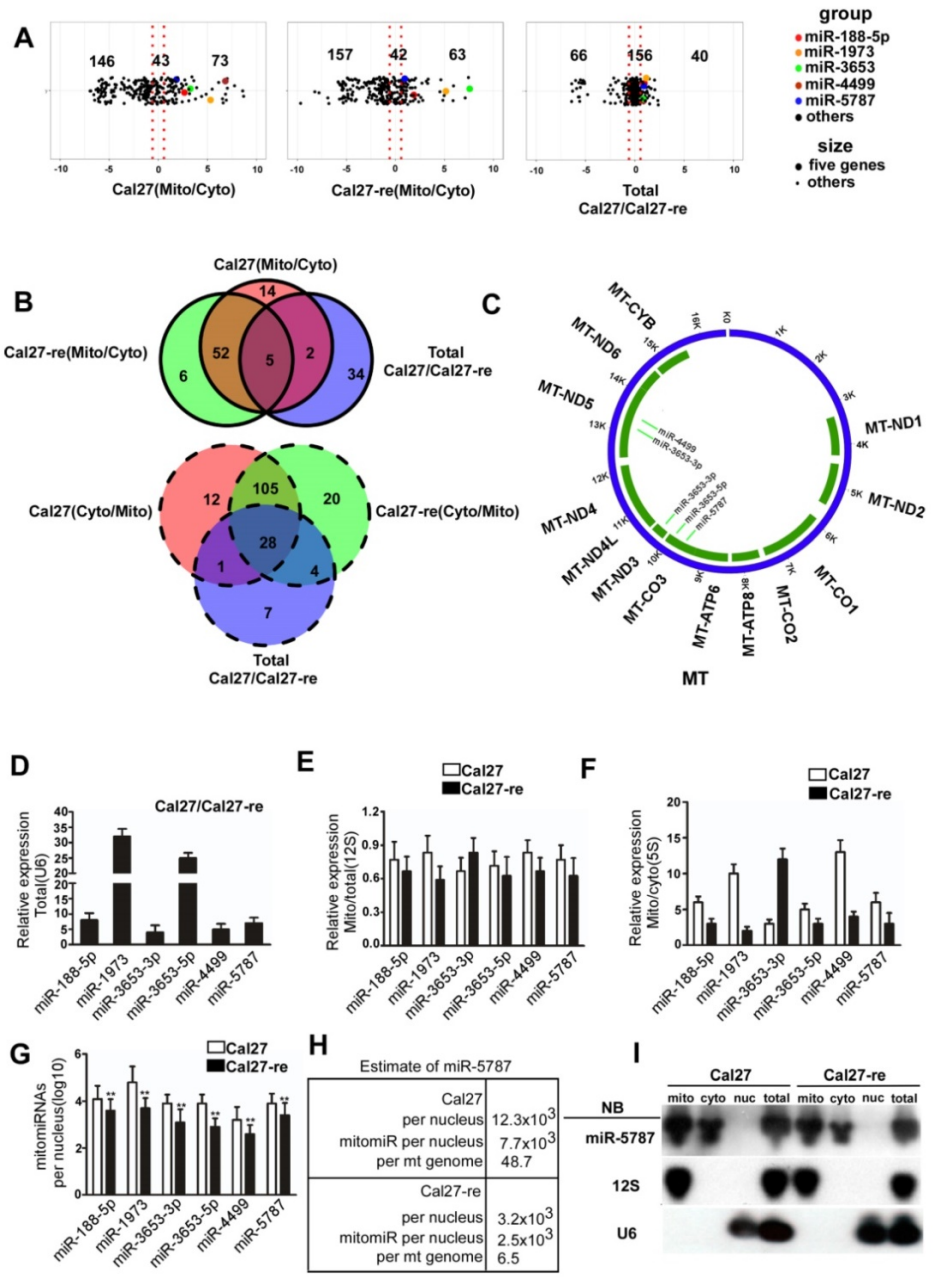
**RNA isolation and identification of miRNA in TSCC-derived mitochondria (A)** Log2-fold change of the relative miRNA probe distribution showed differential miRNA expression in the mitochondrial (Mito)/cytosolic (Cyto) fractions of Cal27 cells (left panel) and the Mito/Cyto fractions of Cal27-re cells (middle panel) as well as miRNAs enriched in Cal-27 cells compared to the levels in Cal27-re cells (right panel). Five mitomiRs downregulated in Cal27-re cells compared with Cal27 cells are highlighted.** (B)** Venn diagram showed mitomiR intersections among three pairs: Cal27 cytosolic miRNAs versus Cal27 mitomiRs, Cal27-re cytosolic miRNAs versus Cal27-re mitomiRs, and Cal27 total miRNAs versus Cal27-re total miRNAs. Five mitomiRs and 28 cytosolic miRNAs decreased in Cal27-re cells compared to the levels in Cal27 cells were identified. **(C)** Screen for target genes of mitomiRs. miR-3653-3p might target MT-ND3 or MT-ND5, miR-3653-5p might target MT-CO3, miR-4499 might target MT-ND5, and miR-5787 might target MT-CO3. **(D)** qRT-PCR verified that six mitomiRs were downregulated in Cal27-re cells compared to Cal27 cells. U6 was chosen as an internal control. **(E)** Comparable expression of six mitomiRs in the mitochondrial fractions and the total cell fractions of Cal27 and Cal27-re cells, with 12S rRNA as the internal control. **(F)** qRT-PCR showed that six mitomiRs were located mainly in the mitochondria in both Cal27 and Cal27-re cells, with 5S rRNA as an internal control for both mitochondria and cytoplasm. **(G)** Quantification of six mitomiRs in Cal27 and Cal27-re nuclei. **P<0.001. **(H)** Quantification of miR-5787 in Cal27 and Cal27-re nuclear or mitochondrial genomes. **(I)** Northern blots (NB) demonstrated the intramitochondrial localization of miR-5787. 12S rRNA and U6 were used as specific mitochondrial and nuclear markers, respectively.

We isolated mitochondria from TSCC cells and confirmed high-purity of this organelle at the DNA, RNA, and protein levels (Supplementary Table S2-S3, and Supplementary Figure S1A). Isolated mitochondria are often contaminated with cytoplasmic RNA. To detect the miRNAs that specifically located in the mitochondria, we used RNase T1 (RN) and Micrococcal nuclease (MN) to treat the isolated mitochondria. RN plus MN can degrade both nuclear U6 small nuclear RNA (snRNA) and cytoplasmic GAPDH mRNA except the RNAs located inside the mitochondria such as 12S rRNA and 16S rRNA. However, RNAs inside the mitochondria are protected and became sensitive to nucleases only in the presence of Triton X-100 (Supplementary Figure S1B). We then performed a miRNA array in paired Cal27/Cal27-re cell lines. Initially, 73 mitomiRs and 146 cytosolic miRNAs were detected in Cal27 cells, while 43 miRNAs were detected in both the mitochondria and the cytoplasm of these cells. On the other hand, in Cal27-re cells, 63 mitomiRs and 157 cytosolic miRNAs along with 42 miRNAs were found in both the mitochondria and cytoplasm. Among these miRNAs, 40 mitomiRs were enriched in Cal27 cells compared to Cal27-re cells, while 66 mitomiRs were enriched in Cal27-re cells compared to their parental cells, and 156 mitomiRs had nearly the same expression levels in both cell lines (Figure 1A). When we compared the miRNAs in the cytosol and mitochondria of the paired TSCC cell lines and considered the miRNAs that were differentially expressed, we found that five mitomiRs and 28 cytoplasmic miRNAs were downregulated in Cal27-re cells (Figure 1B).

Furthermore, we analyzed the potential targets of these mitomiRs in the mitochondrial genome (Figure 1C and Supplementary Table 4). Target prediction suggested that miR-3653-3p might target MT-ND3 or MT-ND5, miR-3653-5p might target MT-CO3, miR-4499 might target MT-ND5, and miR-5787 might target MT-CO3. We used quantitative reverse transcription-polymerase chain reaction (qRT-PCR) to confirm the results from mitomiR array. Six mitomiRs were confirmed to be downregulated in Cal27-re cells (Figure 1D), which was consistent with the miRNA array (miR-3653 consists of miR-3653-3p and miR-3653-5p). As 12S rRNA is located specifically in the mitochondria, by normalizing the expression of these miRNAs to that of 12S rRNA, we confirmed that some miRNAs were enriched in mitochondria (Figure 1E). 5S rRNA is expressed in both the cytosol and mitochondria; therefore, the detection of different miRNA levels in the cytosol and mitochondria suggested that these miRNAs were enriched in mitochondria (Figure 1F). As a cancer cell may contain more than one nucleus, we determined the copy number of nuclear DNA (nDNA) by qPCR to calculate the number of mitomiR molecules per nuclear genome (Figure 1G). However, the expression profiles of the six down-regulated mitomiRs in Cal27-re cells were different in the other paired cells, and only miR-4499 did not significantly differ between the Scc-25 and Scc25-re cells (Supplementary Figure S1C).

We confirmed the expression levels of the differentially expressed miRNAs in the mitochondria and detected that miR-5787 was the most decreased mitomiR in chemoresistant cells (Figure 1G). We also determined the ratio of mitochondrial and nuclear DNAs in our cell models, revealing a ratio of 158 in Cal27 cells and 385 in Cal27-re cells (Supplementary Figure S1D) which were consistent with the previous studies that cisplatin-resistant cells had a greater number of mitochondria than parental cells 15, 16. Next, we determined the number of miR-5787 in the mitochondria, revealing 7.7×10^3^ and 2.5×10^3^ mitomiR-5787 per nDNA in Cal27 and Cal27-re cells, respectively. This translated into 7.7×10^3^/158=48.7 mitomiR-5787 per mtDNA in Cal-27 and 2.5×10^3^/385 =6.5 mitomiR-5787 per mtDNA in Cal27-re. Thus, a total amount of 7.7×10^3^/12.3×10^3^=62.6% and 2.5×10^3^/ 3.2×10^3^=78.1% of mitomiR were present in Cal27 and Cal27-re mitochondria, respectively (Figure 1H). As 12S rRNA is in the mitochondria and U6 is expressed specifically in the nucleus, we further confirmed that miR-5787 is located in the mitochondria and more abundant in Cal27 cells than in Cal27-re by Northern blotting (Figure 1I). Fluorescence *in situ* hybridization (FISH) also showed the localization of miR-5787 in the mitochondria (Supplementary Figure S1E). The data from these experiments demonstrated that mitomiRs were present in TSCC cells. Compared to the parental cells, six mitomiRs were downregulated in the chemoresistant cell lines and four of these had mitochondrial targets with miR-5787 showing the most significant downregulation.

### miR-5787 regulates chemoresistance by enhancing mitochondrial OXPHOS in TSCC cells

**Figure 2:**
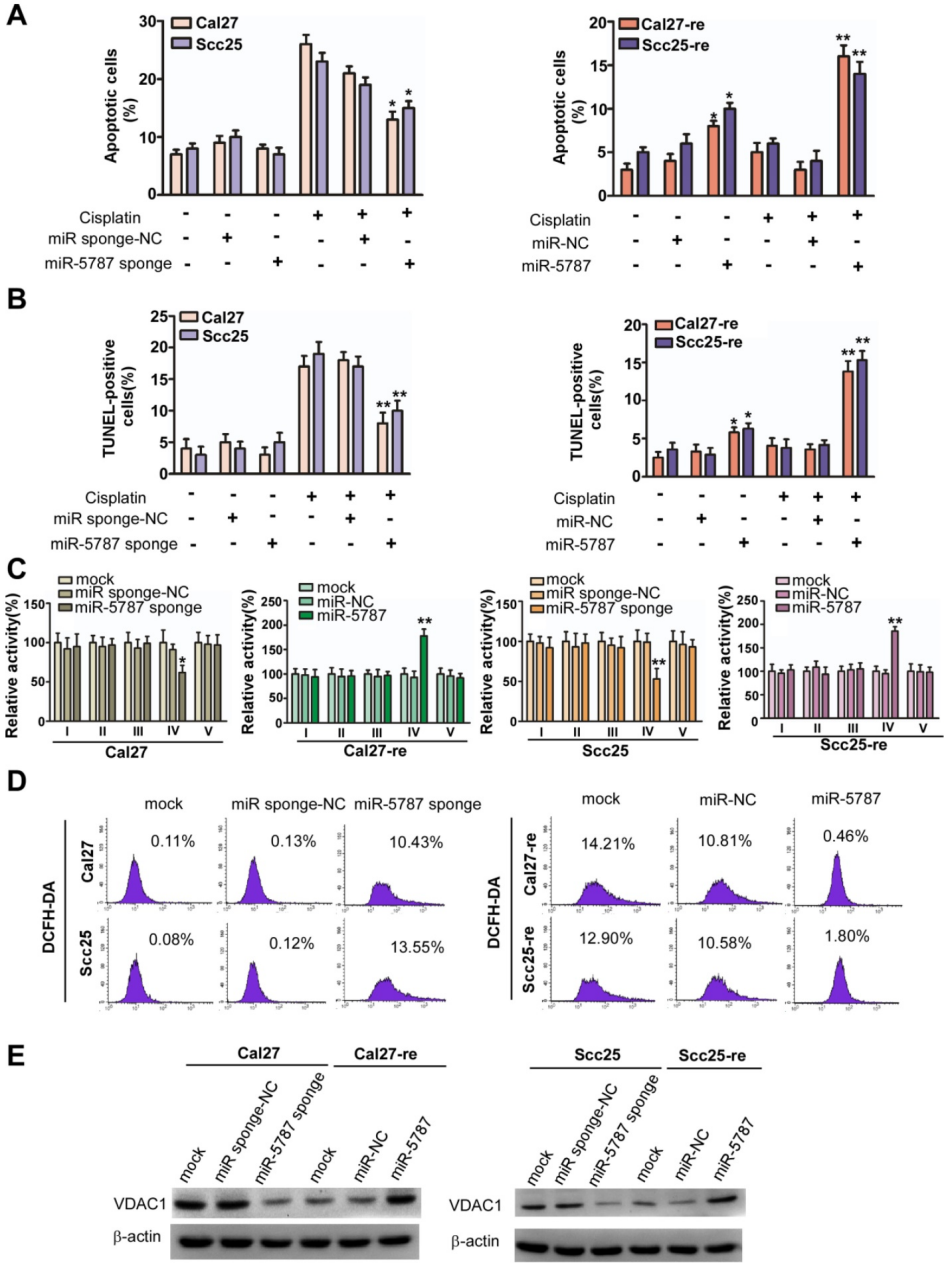
** miR-5787 regulates chemoresistance by enhancing mitochondrial OXPHOS in TSCC cells (A)** Flow cytometry showing Cal27 or Scc25 cells with stable expression of miR-5787 sponge could resist cisplatin-induced apoptosis, while Cal27-re or Scc25-re cells transfected with miR-5787 mimics were sensitive to cisplatin-induced apoptosis. *P<0.01, **P<0.001. **(B)** TUNEL assay showed that Cal27 or Scc25 cells with stable expression of miR-5787 sponge could resist cisplatin-induced apoptosis, while Cal27-re or Scc25-re cells transfected with miR-5787 mimics were sensitive to cisplatin-induced apoptosis. *P<0.01, **P<0.001. **(C)** miR-5787 sponge could downregulate mitochondrial respiratory chain complex IV, while miR-5787 mimics could restore mitochondrial respiratory chain complex IV. *P<0.01, **P<0.001. **(D)** miR-5787 sponge increased ROS generation in Cal27 and Scc25 cells, and miR-5787 mimics decreased ROS generation in Cal27-re and Scc25-re cells. **(E)** Western blots showed the changes in VDAC1 at the protein level. β-actin was used as an internal control.

We examined six mitomiRs for their potential effect on apoptosis in TSCC cells by using flow cytometry and found that only miR-5787 regulated apoptosis (Figure 2A and 2B, Supplementary Figure S2A). miR-5787 has been detected in some cancer cells 17-21, but its functions remain elusive. It is 20 nucleotides in length and originated from chromosome 3. The RNAfold program 22 showed that 55 genomic nucleotides, including the mature miR-5787 sequence, were folded into a stem-loop structure as the premiR-5787. The miR-5787 sequence is conserved in anthropoids and partially conserved in some vertebrates (Supplementary Figure S2B). miRNA mimics are artificial double-stranded miRNA-like RNA fragments designed for gene silencing approaches, while miRNA sponges are RNA transcripts containing multiple high-affinity binding sites that sequester specific miRNAs to reduce their expression and prevent them from interacting with their target mRNAs 13, 23, 24. As the mitochondrion has a double-layer membrane structure, a concentration test showed that 60 nmol miR-5787 mimic could efficiently increase its level in chemoresistant cells, and miR-5787 sponge could downregulate the miR-5787 level in parental cells (Supplementary Figure S2C and S2D).

In Cal27 and Scc25 TSCC cell lines, we used miRNA sponge to downregulate miR-5787 expression and showed that without cisplatin treatment, downregulation of miR-5787 had almost no effect on apoptosis (Figure 2A). However, miR-5787 downregulation induced fewer apoptotic cells when the TSCC cells were exposed to cisplatin. Overexpression of miR-5787 caused increased apoptosis in Cal27-re and Scc25-re cells with or without cisplatin treatment. The TUNEL assay further confirmed the effect of miR- 5787 on apoptosis in TSCC cells (Figure 2B). CCK-8 assay and modified Boyden chamber assay showed that miR-5787 had almost no effect on the proliferation and metastasis of TSCC cells (Supplementary Figure S2E and S2F).

Since miRNAs can reprogram glucose metabolism to affect the biological function of the tumors 7-9, we hypothesized that mitochondrial miR-5787 might also regulate tumor progression via metabolic pathways. A glucose metabolism PCR array indicated that compared with Cal27-re cells, overall OXPHOS activity was enhanced in parental cells while the overall glycolysis activity was decreased (Supplementary Figure S3A). Interestingly, respiratory complex activities (RCAs) differed between parental TSCC cells and chemoresistant TSCC cells. In Cal27-re cells, RCAs I, III, and IV were decreased compared to those in the Cal27 cells, and RCA I, III, IV, and V were decreased in Scc25-re cells compared to those in the Scc25 cells (Supplementary Figure S3B). Knockdown of miR-5787 reduced RCA IV activity in parental TSCC cells, whereas overexpression of miR-5787 could partially rescue RCA IV activity in chemoresistant cells (Figure 2C). As mitochondria are the main source of ROS, we evaluated ROS generation in TSCC cells and found that ROS was increased in chemoresistant cells compared to the levels in parental cells (Supplementary Figure S3C). Interestingly, knockdown of miR-5787 contributed to ROS generation in Cal27 and Scc25 cells, while overexpression of miR-5787 could inhibit ROS generation in chemoresistant cells (Figure 2D and Supplementary Figure S3D).

Generally, mitochondrial activity may be attenuated if mitochondrial OXPHOS is decreased. We found that VDAC1, an important molecule for mitochondrial activity, was downregulated in Cal27 and Scc25 cells when miR-5787 was knocked down, and upregulated in resistant cells when miR-5787 was overexpressed (Figure 2E), suggesting the mitochondrial activity was increased in parental cells and decreased in resistant cells. Thus, knockdown of miR-5787 contributed to chemoresistance in parental TSCC cells, while overexpression of miR-5787 could partially reverse chemoresistance, possibly due to changes in mitochondrial OXPHOS.

### miR-5787 attenuates aerobic glycolysis in TSCC cells

**Figure 3:**
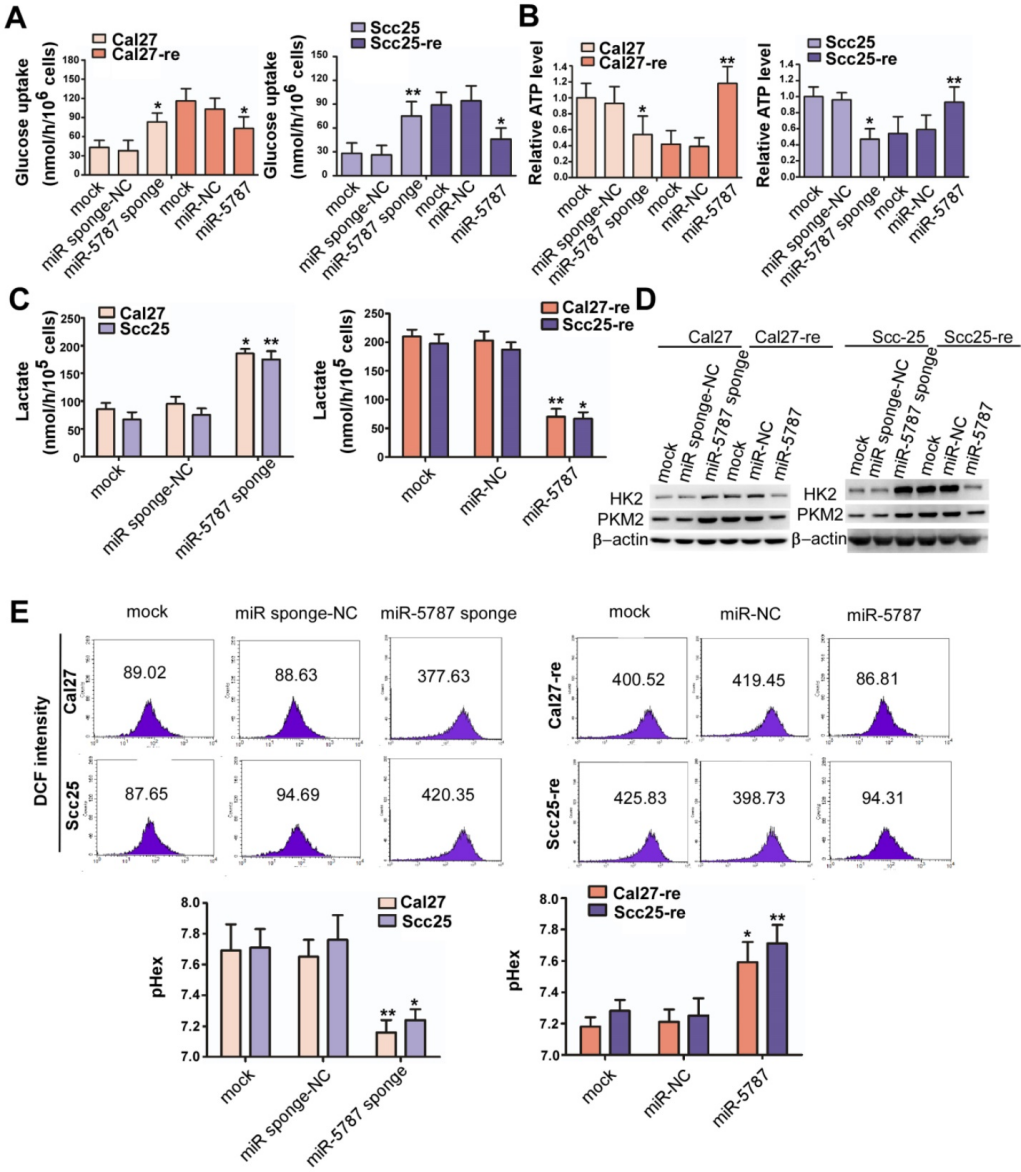
** miR-5787 attenuates aerobic glycolysis in TSCC cells (A)** miR-5787 sponge increased glucose uptake in Cal27 and Scc25 cells, while miR-5787 mimics decreased glucose uptake in Cal27-re and Scc25-re cells. *P<0.01, **P<0.001. **(B)** miR-5787 sponge decreased ATP synthesis in Cal27 and Scc25 cells, while miR-5787 mimics increased ATP synthesis in Cal27-re and Scc25-re cells. *P<0.01, **P<0.001. **(C)** Cal27 and Scc25 cells transfected with miR-5787 sponge generated more lactate, while Cal27-re and Scc25-re cells transfected with miR-5787 mimics generated less lactate. *P<0.01, **P<0.001. **(D)** Western blots showed that the protein levels of HK2 and PKM2 were enhanced when miR-5787 was downregulated in Cal27 and Scc25 cells, while miR-5787 mimics attenuated the protein levels of HK2 and PKM2 in Cal27-re and Scc25-re cells. β-actin was used as an internal control. **(E)** miR-5787 sponge increased pHin and decreased pHex in Cal27 and Scc25 cells, while miR-5787 mimics decreased pHin and increased pHex in Cal27-re and Scc25-re cells. *P<0.01, **P<0.001.

Aerobic glycolysis is a hallmark of cancer that satisfies the increased demand for growth in cancer cells 1. However, aerobic glycolysis levels vary in different cell types. Glycolysis is characterized by increased glucose uptake and reduced adenosine triphosphate (ATP) synthesis. Compared with the parental TSCC cells, glucose uptake was increased by >2-fold while ATP generation was reduced by >2-fold in chemoresistant TSCC cells (Supplementary Figure S4A and S4B). Downregulation of miR-5787 allowed parental TSCC cells to take in more glucose and produce less ATP, while overexpression of miR-5787 in chemoresistant cells decreased glucose uptake and increased ATP synthesis (Figure 3A and 3B). In parental TSCC cells, knockdown of miR-5787 enabled Cal27 and Scc25 cells to produce more lactate, while upregulation of miR-5787 in resistant cells decreased lactate production (Figure 3C). Hexokinase (HK2) and pyruvate kinase isozyme (PKM2) are two critical rate-limiting enzymes in glycolysis 25, 26. In parental TSCC cells, knockdown of miR-5787 could upregulate the expression of HK2 and PKM2. Moreover, overexpression of miR-5787 in resistant cells resulted in the downregulation of the two markers, suggesting the enhancement of aerobic glycolysis in resistant TSCC cells (Figure 3D). Lactate is a component of the culture medium and can change its pH level, and pH-induced physiological drug resistance was reported to play an important role in cisplatin chemotherapy resistance 27-29. We, therefore, examined the effect of miR-5787 on pH changes inside and outside TSCC cells. As expected, knockdown of miR-5787 in Cal27 and Scc25 cells downregulated extracellular pH (pHex), while intracellular pH (pHin) increased; overexpression of miR-5787 in chemoresistant cell lines could increase the pHex and decrease the pHin (Figure 3E). The abnormal intracellular/extracellular pH gradient may account for the cisplatin chemotherapy resistance of TSCC cells. Collectively, these data suggest that a decrease in miR-5787 may change glucose metabolism from OXPHOS to glycolysis, change the pH inside and outside TSCC cells, leading to the chemotherapy resistance of TSCC cells.

### miR-5787 regulates chemoresistance by enhancing the translation of MT-CO3

**Figure 4:**
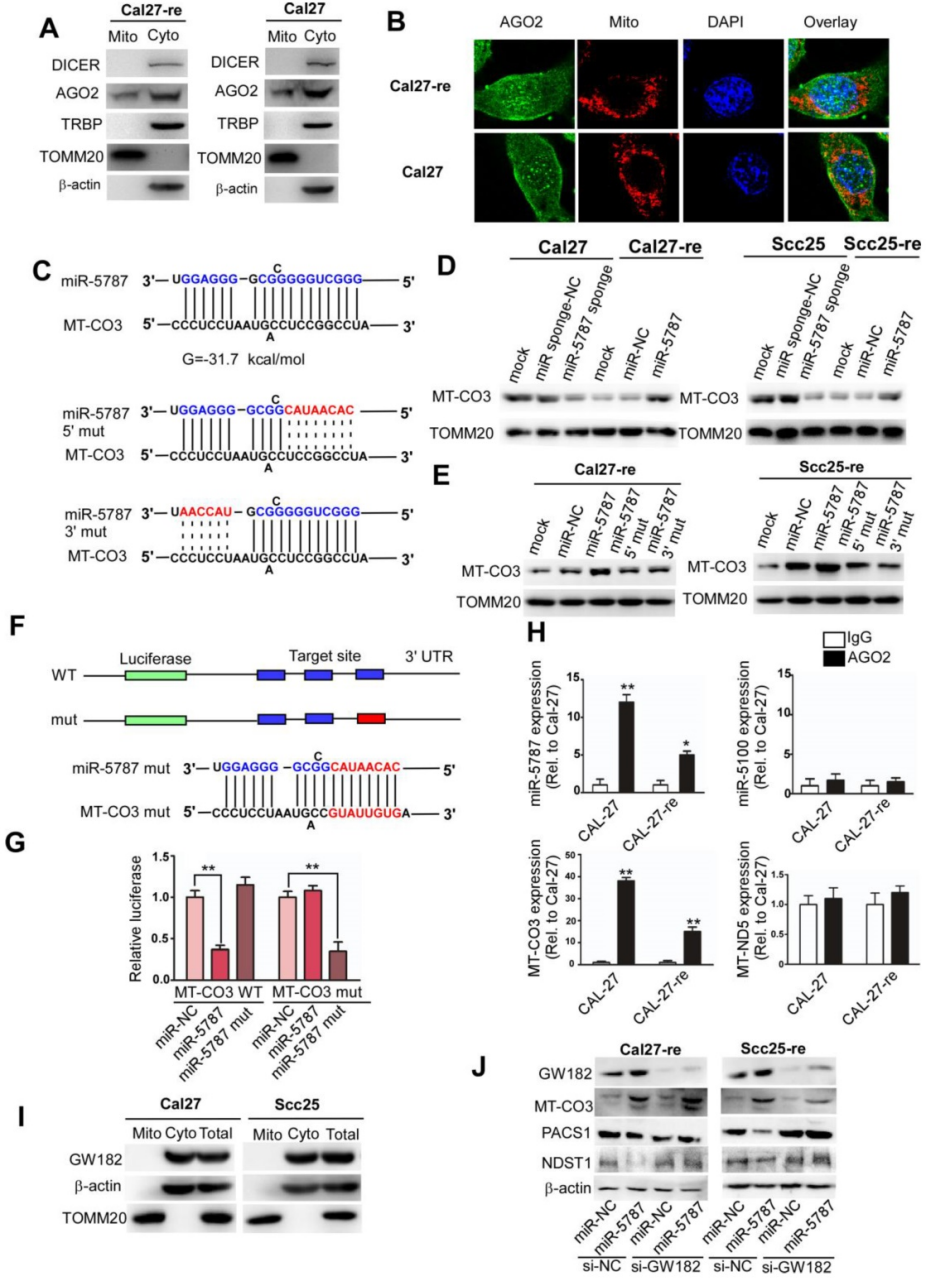
** miR-5787 regulates chemoresistance by enhancing the translation of MT-CO3 (A)** Western blots showed that DICER, TRBP, and β-actin were present only in the cytosol, and TOMM20 was present only in the mitochondria, while AGO2 existed both in the cytosol and the mitochondria. **(B)** Immunofluorescence showed the colocation of AGO2 in the nucleus and mitochondria of Cal27 and Cal27-re cells. **(C)** Target prediction suggested that miR-5787 might target MT-CO3 with the calculated free energy for base pairing as indicated. The 5' and 3' regions of miR-5787 were mutated to further confirm the base pairing. **(D)** Western blots showed that miR-5787 enhanced the expression of MT-CO3 in Cal27 and Scc25 cells, and its downregulation attenuated the expression of MT-CO3 in Cal27-re and Scc25-re cells. TOMM20 was used as an internal control. **(E)** Western blots showed that miR-5787 had almost no effect on the translation of MT-CO3 when its 5' or 3' region was mutated. TOMM20 was used as an internal control. **(F, G)** miR-5787 repressed a cytoplasmic reporter containing three miR-5787 target sites from MT-CO3. The mutant miR-5787 site in the reporter lost its effect, which could be restored with the corresponding mutant miR-5787, which reestablished the required base-pairing interactions. **P<0.001. **(H)** RNA immunoprecipitation showed that miR-5787 but not miR-5100 might interact with MT-CO3, and miR-5787 interacted with MT-CO3 but not MT-ND5. *P<0.01, **P<0.001. **(I)** Western blots show that GW182 existed only in the cytosol and was absent from the mitochondria. β-actin was used as a cytosolic control, and TOMM20 was used as a mitochondrial control.** (J)** Effects of miR-5787 on the translation of MT-CO3, PACS1 and NDST1 with the miRNA machinery selectively inactivated by knocking down GW182 in the cytoplasm.

Cytoplasmic miRNA can imperfectly bind to mRNA transcripts with complementary sequences, usually in the 3'UTR, inhibiting mRNA translation and/or inducing mRNA degradation 6. RNA-induced silencing complex (RISC) proteins are vital for the effects of miRNA, Western blots and immunofluorescence analysis indicated that only the key member AGO2 was present in the mitochondria (Figure 4A and 4B). However, DICER and TRBP, which are involved in the processing of mature miRNA, were scarce in the mitochondria. To distinguish mitochondrial miR-5787 from cytosolic miR-5787, we traced the origin of this mitomiR by qRT-PCR. We observed that premiR-5787 was located mainly in the cytosol and was barely detected in the mitochondria or nucleus (Supplementary Figure S5A). These findings indicated that mitomiRs may be generated in the same way as cytoplasmic miRNAs, which originate in the nucleus, mature in the cytosol and ultimately translocate into the mitochondria.

Bioinformatic analysis indicated that miR-5787 might interact with MT-CO3 (Figure 4C). qRT-PCR suggested that the RNA levels of MT-ND2, MT-CO1, MT-ND4, MT-ND5, and MT-CYB were decreased in Cal27-re compared to those in Cal27, and the RNA levels of MT-CO1, MT-CO2, MT-ND3, MT-ND4, and MT-CYB were decreased in Scc25-re compared to those in Scc25 (Supplementary Figure S5B). Downregulation of miR-5787 in parental TSCC cells or its overexpression in chemoresistant TSCC cells had no effect on MT-CO3 RNA levels (Supplementary Figure S5C). However, downregulation of miR-5787 could decrease MT-CO3 expression, and the overexpression of miR-5787 could increase MT-CO3 expression at the protein level (Figure 4D), implying its regulation at the posttranscriptional level. Mutations in the 3' or 5' regions of miR-5787 could abolish this effect on MT-CO3 protein levels (Figure 4C, E). To confirm whether miR-5787 targets MT-CO3 in TSCC cells, we cloned the target sites into the 3'UTR of a luciferase reporter. Overexpression of the wild type miR-5787 decreased the reporter activity, while the mutant miR-5787 lost this effect, but the repressive effect recurred when we changed the target sequences to be paired with mutant miR-5787 in the reporter (Figure 4F, G). RNA immunoprecipitation in the mitochondria confirmed that miR-5787 but not miR-5100 (enriched in the cytosol) might target MT-CO3 (Figure 4H).

To eliminate the effects of cytosolic targets of miR-5787 on chemoresistance of TSCCs, we searched for cytosolic targets and evaluated their effects. Based on target prediction (miRDB 30 and TargetScan 31) and top target ranking, we hypothesized that miR-5787 may target the 3'UTRs of PACS1 and NDST1 (Supplementary Figure S5D). Knockdown of miR-5787 in Cal27 and Scc25 cells enhanced the translation of PACS1 and NDST1, while overexpression of miR-5787 in Cal27-re and Scc25-re cells reversed this effect (Supplementary Figure S5E). To determine the direct effect of miR-5787 on the 3'UTRs of the PACS1 and NDST1 mRNAs, we cloned the 3'UTRs of PACS1 (PACS1-wt-3'UTR) and NDST1 (NDST1-wt-3'UTR) into the firefly luciferase reporter construct pG13. Mutant 3'UTRs were also constructed for PACS1 (PACS1-mut-3'UTR) and NDST1 (NDST1-mut-3'UTR) as controls. We transfected these constructs into HEK293T cells, along with a scrambled short RNA sequence or miR-5787 and evaluated luciferase activity. Upregulation of miR-5787 significantly decreased the activity of wild-type 3'UTR but had almost no effect on the reporter activity of mutant 3'UTR (Supplementary Figure S5F). Next, siRNAs were applied to determine the effects of knockdown of PACS1 and NDST1 on apoptosis and the chemoresistance of TSCC cells (Supplementary Figure S5G). Flow cytometry and TUNEL assays showed no effect on apoptosis in TSCC cells which excluded the possibility that miR-5787 targets PACS1 and NDST1 to regulate the chemoresistance of TSCC cells (Supplementary Figure S5H and S5I). However, knockdown of MT-CO3 decresed the RCA IV activity, increased the glucose uptake, decreased the ATP synthesis and increased the lactate generation, so the attenuation of OXPHOS and the enhancement of aerobic glycolysis eventually led to chemoresistance (Supplementary Figure S6A-6E). Altogether, these results suggested that miR-5787 acted through the mitochondrial target MT-CO3 rather than its cytosolic targets PACS1 and NDST1. Furthermore, Zhang et al. showed that GW182, a key factor for RISC function, was absent in the mitochondria, which might explain how mitomiRs could enhance rather than downregulate the expression of target genes 13. We assessed GW182 expression in TSCC cells and obtained comparable results (Figure 4I). GW182 knockdown prevented miR-5787-induced translational repression of its cytoplasmic targets PACS1 and NDST1 in two TSCC cell lines (Figure 4J). However, GW182 RNAi- treated TSCC cells exhibited enhanced MT-CO3 translation by miR-5787 mimics (Figure 4J). These data demonstrated that miR-5787 targeted MT-CO3 upregulating its expression at the protein level and partially reversing chemoresistance of TSCC cells.

### Effects of miR-5787 on the chemoresistance of TSCC cells in xenografts

**Figure 5:**
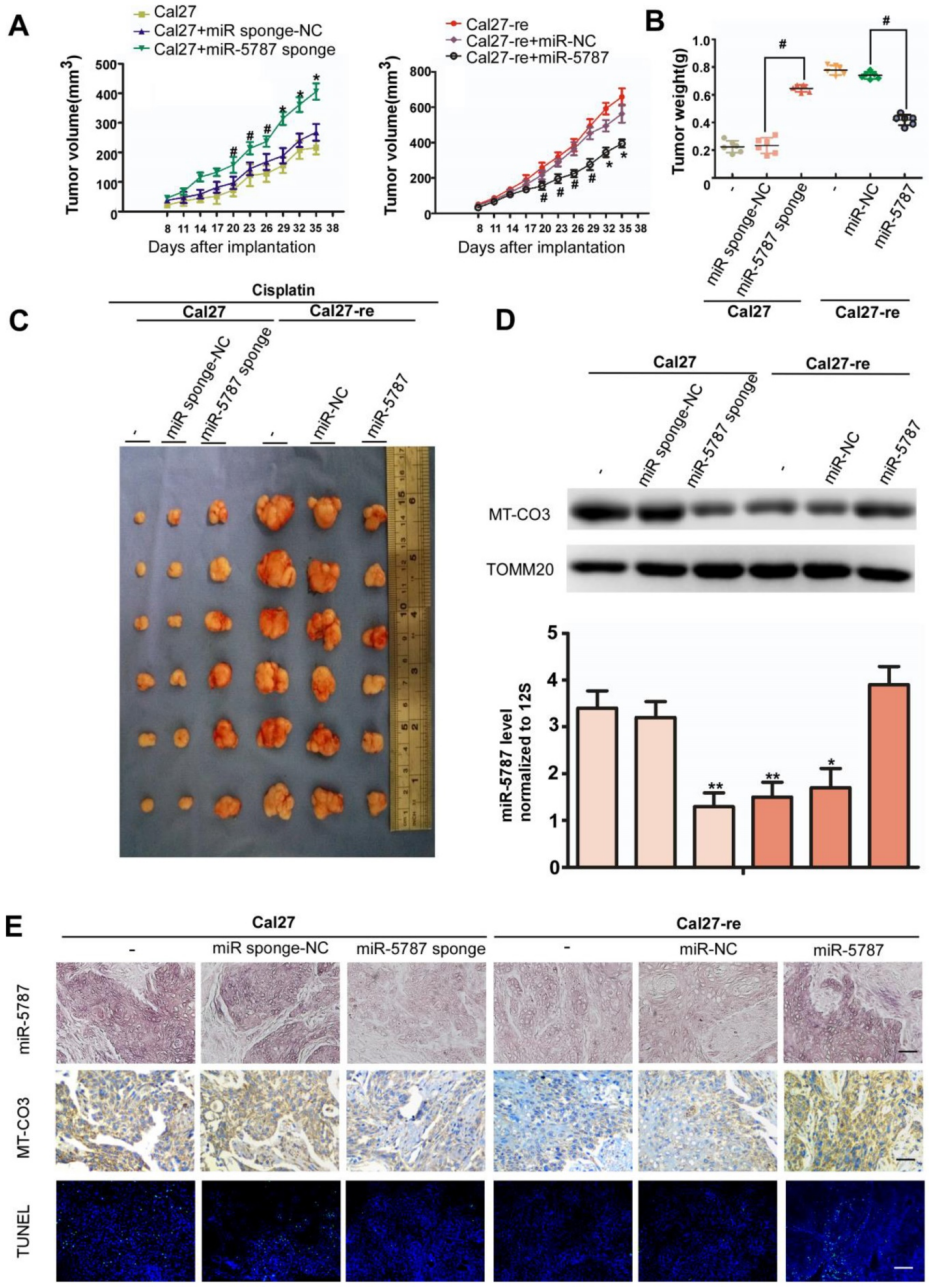
** miR-5787 regulates the chemoresistance of TSCC cells in xenografts in BALB/c-nu mice. (A)** Tumor growth curves for Cal27 and Cal27-re tumors treated with cisplatin. miR-5787 was stably downregulated in Cal27 cells, while miR-5787 was stably upregulated in Cal27-re cells; Cal27 or Cal27-re cells without any treatment or Cal27 or Cal27-re cells that were stably transfected with miR-control (NC) were used as control groups. n=6 in each group. #*P*<0.05, *P<0.01.** (B)** Tumor weights showed the effects of miR-5787 on the indicated two groups. **(C)** Tumor xenograft images on day 35.** (D)** MT-CO3 expression was analyzed using Western blots (upper), and miR-5787 expression was determined by qRT-PCR (below). *P<0.01**, *P*<0.001. **(E)** miR-5787 and MT-CO3 were expressed in xenografts in each group, and apoptosis was detected by TUNEL assays. miR-5787 and MT-CO3 expression was analyzed by *in situ* hybridization and immunohistochemistry, respectively (200×). n=24 slices from six xenograft tumors per group. The scale bar for *in situ* hybridization and immunohistochemistry represents 40 μm, and the scale bar for TUNEL assays represents 20 μm.

To further explore the effect of miR-5787 on TSCC cells *in vivo*, we performed xenograft experiments in BALB/c-nu mice. Parental TSCC cells in which miR-5787 was stably knocked down showed less apoptosis when exposed to cisplatin, as indicated by tumor growth curves, weight, and volume (Figure 5A, 5B and 5C). On the other hand, chemoresistant TSCC cells in which miR-5787 was upregulated became sensitive to cisplatin, as shown by increased apoptosis, lighter weight, and smaller xenograft sizes (Figure 5A, 5B and 5C). Xenografts with high expression of miR-5787 showed enhanced expression of MT-CO3 and were more sensitive to cisplatin therapy compared to cells with normal miR-5787 expression (Figure 5D, 5E) which was consistent with the results obtained *in vitro*. TUNEL staining showed that xenografts with high expression of miR-5787 and MT-CO3 had more apoptotic cells (Figure 5E). These results further confirmed the effects of miR-5787 on the chemoresistance of TSCC cells *in vivo*.

### Low expression of miR-5787 in TSCC cells predicts poor patient survival outcome

**Figure 6:**
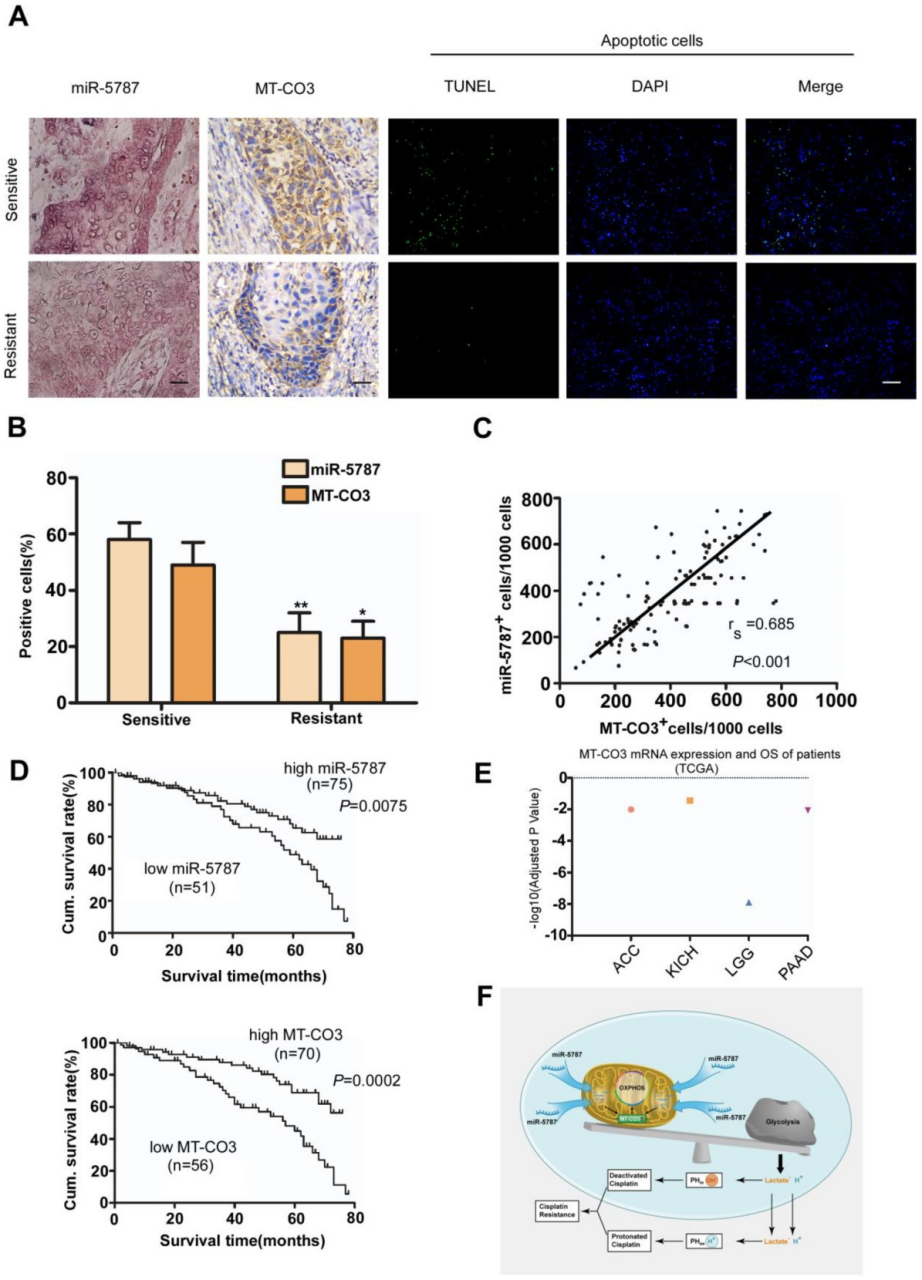
** miR-5787 and MT-CO3 expression correlates with cisplatin chemoresistance and patient survival in TSCC patients. (A)** miR-5787 and MT-CO3 expression and apoptosis was evaluated in cisplatin-sensitive and cisplatin-resistant TSCC patients. miR-5787 was analyzed using *in situ* hybridization, and MT-CO3 expression was analyzed via immunohistochemistry (200×). Apoptosis was detected using the TUNEL assay. The scale bar for *in situ* hybridization and immunohistochemistry represents 40 μm, and the scale bar for TUNEL assays represents 20 μm. **(B)** Quantification of miR-5787 and MT-CO3 expression in cisplatin-sensitive versus cisplatin-resistant TSCC patients. *P<0.01, **P<0.001. **(C)** The correlation between miR-5787 and MT-CO3 expression in TSCC patients was analyzed by Spearman order correlation. **(D)** Kaplan-Meier survival curves for TSCC patients were plotted for miR-5787 and MT-CO3 expression, and survival differences were analyzed using a log-rank test. **(E)** Association of MT-CO3 mtRNA expression levels with patients' overall survival in multiple types of human cancers based on TCGA RNA sequencing data. (ACC, adrenocortical carcinoma; KICH, kidney chromophobe; LGG, low-grade glioma; PAAD, pancreatic adenocarcinoma). **(F)** Graphic model of this study.

To determine the clinical significance of miR-5787 and its target gene MT-CO3, we performed a retrospective analysis on TSCC samples from 126 patients. The clinical data suggested no significant correlation between either miR-5787 or MT-CO3 and age, sex, clinical stage, or lymph node metastasis (Table 1). However, miR-5787 and MT-CO3 levels were closely associated with chemoresistance status of the patients. ISH and IHC demonstrated that cisplatin-sensitive TSCC patients had high expression of miR-5787 and MT-CO3 compared to cisplatin- resistant patients (Figure 6A and 6B). Furthermore, Spearman order correlation analysis indicated that miR-5787 was positively associated with MT-CO3 (Figure 6C). We also evaluated the correlations between miR-5787 or MT-CO3 expression with patients' overall survival (OS). Univariate Cox regression analysis indicated that TSCC patients with low miR-5787 expression levels or low MT-CO3 levels had a shorter OS (Table 1 and Figure 6D). The cumulative survival rates at 60 months were 70.67% and 71.43% in patients with high miR-5787 and MT-CO3 expression, respectively, while only 37.25% and 39.29% in patients with low miR-5787 and MT-CO3 expression, respectively (Table 1). Furthermore, multivariate Cox regression analysis revealed that low-level expression of miR-5787 and MT-CO3 were independent prognostic factors for poorer OS in patients with TSCC (Table 2). Interestingly, TCGA RNA sequencing data also identified an association of higher MT-CO3 expression levels with increased overall survival in four cancer types, including adrenocortical carcinoma (ACC), kidney chromophobe (KICH), low-grade glioma (LGG), and pancreatic adenocarcinoma (PAAD) (Figure 6E). Collectively, these data suggested that miR-5787 and its mitochondrial target MT-CO3 correlated with cisplatin sensitivity and patient OS in patients with TSCC. A graphic model of this study is displayed in Figure 6F.

## Discussion

The mitochondrion has been regarded as a cellular powerhouse. But recently, accumulating studies revealed its crucial role in other essential biological processes, such as apoptosis, calcium homeostasis, and the production of ROS 32-34. Cancer cells possess a distinctive characteristic related to glucose metabolism, described as the “Warburg effect”. Thus, as an organelle of metabolism, the mitochondria may exert specific effects on glucose metabolism in malignant cells. Although miRNAs are typically found in the cytoplasm, recent studies showed that miRNAs also exist in the mitochondria 10-14, 35, 36. In this study, we showed that among the mitomiRs found in the mitochondria of TSCC cells, miR-5787 was downregulated in chemoresistant cells, potentially playing a role in glucose metabolism and chemoresistance of TSCC cells.

miRNAs can affect glucose metabolism in tumor cells by directly enhancing or attenuating glycolysis and impacting the development and/or progression of tumors. In hepatocellular carcinoma (HCC), TARDBP-PFKP-miR-520 axis enhanced glycolysis to inhibit the growth of HCC cells 37. Recent studies revealed that miRNAs, including miR-124, miR-137, miR-340, miR-143, and miR-155, regulated glycolysis by directly targeting the 3'UTR region of HK2 and PKM2, thereby affecting cell proliferation, apoptosis, and metastasis 38, 39. In this study, we observed that mitomiRs could also regulate glucose metabolism. The downregulation of mitochondrial miR-5787 attenuated OXPHOS and enhanced aerobic glycolysis.

Numerous miRNAs are involved in chemoresistance of cancer cells which is one of the main causes of tumor recurrence. Some miRNAs, such as miR-141, miR-200c, and miR-21, contribute to the chemoresistance of cancer cells 40-42. For instance, in our previous studies, we have shown that miR-593-5p targeted the MFF and miR-483-5p targeted FIS1 to regulate chemoresistance 43, 44. On the contrary, some miRNAs have been reported to sensitize cancer cells to chemotherapy 45-49. However, the function of mitomiRs in the regulation of chemosensitivity has remained mostly unexplored. In this study, for the first time, we demonstrated that mitomiR-5787 could regulate cisplatin resistance of TSCC. We showed that knockdown of miR-5787 could down regulate the expression of VDAC1 and up regulate the expression of PKM2 and HK2, resulting in the attenuation of OXPHOS and the enhancement of aerobic glycolysis. Other studies have indicated that overexpression of VDAC1 could induce apoptosis 50, inhibiting expression of PKM2 could increase cisplatin sensitivity of osteosarcoma stem cells and esophageal squamous cell carcinoma cells 51,52, and up-regulation of HK2 conferred resistance to cisplatin in ovarian cancer cells, which were in accordance with our results 53. Furthermore, Numerous studies revealed that enhancement of aerobic glycolysis contributes to cisplatin resistance while stimulation of OXPHOS can reverse cisplatin resistance 54,55. We demonstrated that miR-5787 regulated cisplatin induced apoptosis through manipulation of OXPHOS and aerobic glycolysis.

miRNAs have been demonstrated to exist in mitochondria, but their significance remains obscure. Recently, Das et al. revealed the role of mitomiRs in myocardial pathophysiology 12. These authors showed that the nucleus-originated miR-181c could inhibit translation of MT-COX1 to regulate mitochondrial energy metabolism. Another study revealed that miR-1 efficiently entered mitochondria to stimulate the translation of specific mitochondrial genome-encoded transcripts 13. Our present study demonstrated mitochondrial miR-5787 targeted MT-CO3 and enhanced rather than repress its translation to reverse chemoresistance.

The human mitochondrial genome consists of a 16-kb circular double-stranded DNA that encodes 13 proteins, including four enzyme complexes (complex I, III, and IV and ATPase synthetase) of the respiratory chain, as well as 2 rRNAs and 22 tRNAs 56. Changes in mitochondrial DNA have been reported in cancer cells 57. However, previous studies focused mainly on mtDNA polymorphisms 58. In this study, using a cancer model, we showed that miR-5787 enhanced the translation of MT-CO3 in a posttranscriptional manner.

The pHex value of malignant tumor cells is lower than the corresponding pHin value, and the intracellular/extracellular pH gradient is opposite to that of normal cells 59. Low pH values cause negative effects on tumor cells; thus, Na^+^/H^+^ exchange proteins in the cytomembrane of tumor cells are upregulated 60, 61. pH-induced physiological drug resistance is clinically significant. When the environmental pH value is lower than the pKa of the drug, the OH^-^ of cisplatin and other weakly alkaline drugs is neutralized by H^+^ from the environment. Simultaneously, the high pHin value prevents the activation of cisplatin inside the cells resulting in chemotherapy resistance. We confirmed that pHin is high and pHex is low in chemoresistant cells, and the abnormal intracellular/extracellular pH gradient may partially explain the chemotherapy resistance in TSCC.

In summary, the results of our study revealed that when miR-5787 concentration is low in the mitochondria, the translation of MT-CO3 is inhibited, resulting in the attenuation of OXPHOS and enhancement of glycolysis. More lactate is then produced causing cisplatin to lose efficacy in a low-pH environment and developing cisplatin resistance.

## Figures and Tables

**Table 1:** Correlation among clinicopathological status and the expression of miR-5787, MT-CO3 in TSCC patients.

Characteristic	miR-5787 expression		*P*	MT-CO3 expression		*P*
	Low, n (%)	High, n (%)		Low, n (%)	High, n (%)	
Sex			0.979			0.119
Male	28 (40.5)	41 (59.5)		35 (50.7)	34 (49.3)	
Female	23 (40.3)	34 (59.7)		21 (36.8)	36 (63.2)	
Age			0.291			0.262
<50	21 (46.7)	24 (53.3)		23 (51.1)	22 (48.9)	
≥50	30 (37.0)	51 (63.0)		33 (46.5)	48 (53.5)	
Clinical stage			0.699			0.293
III	31 (41.9)	43 (58.1)		30 (40.5)	44 (59.5)	
IV	20 (38.5)	32 (61.5)		26 (50.0)	26 (50.0)	
Node metastasis			0.917			0.550
N0	27 (40.9)	39 (59.1)		31 (47.0)	35 (53.0)	
N+	24 (40.0)	36 (60.0)		25 (41.7)	35 (58.3)	
Cisplatin			<0.001			<0.001
Sensitive	13 (18.1)	59 (81.9)		11 (15.3)	61 (84.7)	
Resistant	38 (70.4)	16 (29.6)		45 (83.3)	9 (16.7)	
Status			<0.001			<0.001
Survival	19 (26.4)	53 (73.6)		22 (30.6)	50 (69.4)	
Death	32 (59.3)	22 (40.7)		34 (63.0)	20 (37.0)	

**Table 2:** Univariate and multivariate analysis of factors associated with overall survival of patients with TSCC.

Variable	Cases number	HR(95%CI)	*P*
**Univariate analysis**			
Sex Male vs Female	69/57	0.851(0.464-1.562)	0.557
Age(years) <50 vs ≥50	45/81	0.759(0.519-1.109)	0.692
Node metastasis N0 vs N+	66/60	1.383(1.015-1.885)	0.036
Clinical stage III VS IV	74/52	2.742(1.658-4.535)	<0.001
Cisplatin Sensitive vs Resistant	72/54	0.695(0.561-0.861)	0.032
miR-5787 Low vs High	51/75	1.976(1.359-2.873)	0.008
MT-CO3 Low vs High	56/70	1.568(1,126-2.184)	0.017
**Multivariate analysis**			
Node metastasis N0 vs N+	66/60	1.836(1.178-2.861)	0.005
Clinical stage III vs IV	74/52	2.563(1.689-3.889)	<0.001
Cisplatin Sensitive vs Resistant	72/54	0.694(0.569-0.846)	0.039
miR-5787 Low vs High	51/75	1.543(1.106-2.153)	0.015
MT-CO3 Low vs High	56/70	1.862(1.395-2.485)	0.011

## References

[1] Warburg O (1956). On respiratory impairment in cancer cells. Science.

[2] Kim JW, Dang CV (2006). Cancer's molecular sweet tooth and the Warburg effect. Cancer Res.

[3] Viale A, Pettazzoni P, Lyssiotis CA, Ying H, Sanchez N, Marchesini M (2014). Oncogene ablation-resistant pancreatic cancer cells depend on mitochondrial function. Nature.

[4] Lagadinou ED, Sach A, Callahan K, Rossi RM, Neering SJ, Minhajuddin M (2013). BCL-2 inhibition targets oxidative phosphorylation and selectively eradicates quiescent human leukemia stem cells. Cell Stem Cell.

[5] Suen DF, Norris KL, Youle RJ (2008). Mitochondrial dynamics and apoptosis. Genes Dev.

[6] Berezikov E (2011). Evolution of microRNA diversity and regulation in animals. Nat Rev Genet.

[7] Fong MY, Zhou W, Liu L, Alontaga AY, Chandra M, Ashby J (2015). Breast-cancer-secreted miR-122 reprograms glucose metabolism in premetastatic niche to promote metastasis. Nat Cell Biol.

[8] Eichner LJ, Perry MC, Dufour CR, Bertos N, Park M, St-Pierre J (2010). miR-378(*) mediates metabolic shift in breast cancer cells via the PGC-1beta/ERRgamma transcriptional pathway. Cell Metab.

[9] Nie H, Li J, Yang XM, Cao QZ, Feng MX, Xue F (2015). Mineralocorticoid receptor suppresses cancer progression and the Warburg effect by modulating the miR-338-3p-PKLR axis in hepatocellular carcinoma. Hepatology.

[10] Kren BT, Wong PY, Sarver A, Zhang X, Zeng Y, Steer CJ (2009). MicroRNAs identified in highly purified liver-derived mitochondria may play a role in apoptosis. RNA Biol.

[11] Bian Z, Li LM, Tang R, Hou DX, Chen X, Zhang CY (2010). Identification of mouse liver mitochondria-associated miRNAs and their potential biological functions. Cell Res.

[12] Das S, Ferlito M, Kent OA, Fox-Talbot K, Wang R, Liu D (2012). Nuclear miRNA regulates the mitochondrial genome in the heart. Circ Res.

[13] Zhang X, Zuo X, Yang B, Li Z, Xue Y, Zhou Y (2014). MicroRNA directly enhances mitochondrial translation during muscle differentiation. Cell.

[14] Li H, Zhang X, Wang F, Zhou L, Yin Z, Fan J (2016). MicroRNA-21 Lowers Blood Pressure in Spontaneous Hypertensive Rats by Upregulating Mitochondrial Translation. Circulation.

[15] Zhang Y, Maurizi MR (2016). Mitochondrial ClpP activity is required for cisplatin resistance in human cells. Biochim Biophys Acta.

[16] Jeon JH, Kim DK, Shin Y, Kim HY, Song B, Lee EY (2016). Migration and invasion of drug-resistant lung adenocarcinoma cells are dependent on mitochondrial activity. Exp Mol Med.

[17] Castro-Magdonel BE, Orjuela M, Camacho J, Garcia-Chequer AJ, Cabrera-Munoz L, Sadowinski-Pine S (2017). miRNome landscape analysis reveals a 30 miRNA core in retinoblastoma. Bmc Cancer.

[18] Sana J, Busek P, Fadrus P, Besse A, Radova L, Vecera M (2018). Identification of microRNAs differentially expressed in glioblastoma stem-like cells and their association with patient survival. Sci Rep.

[19] Fu F, Jiang W, Zhou L, Chen Z (2018). Circulating Exosomal miR-17-5p and miR-92a-3p Predict Pathologic Stage and Grade of Colorectal Cancer. Transl Oncol.

[20] Mullany LE, Herrick JS, Wolff RK, Stevens JR, Slattery ML (2016). Association of cigarette smoking and microRNA expression in rectal cancer: Insight into tumor phenotype. Cancer Epidemiol.

[21] Luo Y, Wen X, Wang L, Gao J, Wang Z, Zhang C (2016). Identification of MicroRNAs Involved in Growth Arrest and Apoptosis in Hydrogen Peroxide-Treated Human Hepatocellular Carcinoma Cell Line HepG2. Oxid Med Cell Longev.

[22] Hofacker IL (2003). Vienna RNA secondary structure server. Nucleic Acids Res.

[23] Chekulaeva M, Filipowicz W (2009). Mechanisms of miRNA-mediated post-transcriptional regulation in animal cells. Curr Opin Cell Biol.

[24] Sun L, Yao Y, Liu B, Lin Z, Lin L, Yang M (2012). MiR-200b and miR-15b regulate chemotherapy-induced epithelial-mesenchymal transition in human tongue cancer cells by targeting BMI1. Oncogene.

[25] Guo W, Qiu Z, Wang Z, Wang Q, Tan N, Chen T (2015). MiR-199a-5p is negatively associated with malignancies and regulates glycolysis and lactate production by targeting hexokinase 2 in liver cancer. Hepatology.

[26] Chen M, Sheng XJ, Qin YY, Zhu S, Wu QX, Jia L (2019). TBC1D8 Amplification Drives Tumorigenesis through Metabolism Reprogramming in Ovarian Cancer. Theranostics.

[27] Raghunand N, He X, van Sluis R, Mahoney B, Baggett B, Taylor CW (1999). Enhancement of chemotherapy by manipulation of tumour pH. Br J Cancer.

[28] Luciani F, Spada M, De Milito A, Molinari A, Rivoltini L, Montinaro A (2004). Effect of proton pump inhibitor pretreatment on resistance of solid tumors to cytotoxic drugs. J Natl Cancer Inst.

[29] Dechant R, Binda M, Lee SS, Pelet S, Winderickx J, Peter M (2010). Cytosolic pH is a second messenger for glucose and regulates the PKA pathway through V-ATPase. Embo J.

[30] Wong N, Wang X (2015). miRDB: an online resource for microRNA target prediction and functional annotations. Nucleic Acids Res.

[31] Lewis BP, Shih IH, Jones-Rhoades MW, Bartel DP, Burge CB (2003). Prediction of mammalian microRNA targets. Cell.

[32] Renault TT, Floros KV, Elkholi R, Corrigan KA, Kushnareva Y, Wieder SY (2015). Mitochondrial shape governs BAX-induced membrane permeabilization and apoptosis. Mol Cell.

[33] Chen X, Zhang X, Kubo H, Harris DM, Mills GD, Moyer J (2005). Ca2+ influx-induced sarcoplasmic reticulum Ca2+ overload causes mitochondrial-dependent apoptosis in ventricular myocytes. Circ Res.

[34] Scialo F, Sriram A, Fernandez-Ayala D, Gubina N, Lohmus M, Nelson G (2016). Mitochondrial ROS Produced via Reverse Electron Transport Extend Animal Lifespan. Cell Metab.

[35] Barrey E, Saint-Auret G, Bonnamy B, Damas D, Boyer O, Gidrol X (2011). Pre-microRNA and mature microRNA in human mitochondria. Plos One.

[36] Bandiera S, Ruberg S, Girard M, Cagnard N, Hanein S, Chretien D (2011). Nuclear outsourcing of RNA interference components to human mitochondria. Plos One.

[37] Park YY, Kim SB, Han HD, Sohn BH, Kim JH, Liang J (2013). Tat-activating regulatory DNA-binding protein regulates glycolysis in hepatocellular carcinoma by regulating the platelet isoform of phosphofructokinase through microRNA 520. Hepatology.

[38] Jiang S, Zhang LF, Zhang HW, Hu S, Lu MH, Liang S (2012). A novel miR-155/miR-143 cascade controls glycolysis by regulating hexokinase 2 in breast cancer cells. Embo J.

[39] Peschiaroli A, Giacobbe A, Formosa A, Markert EK, Bongiorno-Borbone L, Levine AJ (2013). miR-143 regulates hexokinase 2 expression in cancer cells. Oncogene.

[40] van Jaarsveld MT, Helleman J, Boersma AW, van Kuijk PF, van Ijcken WF, Despierre E (2013). miR-141 regulates KEAP1 and modulates cisplatin sensitivity in ovarian cancer cells. Oncogene.

[41] Hamano R, Miyata H, Yamasaki M, Kurokawa Y, Hara J, Moon JH (2011). Overexpression of miR-200c induces chemoresistance in esophageal cancers mediated through activation of the Akt signaling pathway. Clin Cancer Res.

[42] Au YC, Co NN, Tsuruga T, Yeung TL, Kwan SY, Leung CS (2016). Exosomal transfer of stroma-derived miR21 confers paclitaxel resistance in ovarian cancer cells through targeting APAF1. Nat Commun.

[43] Fan S, Chen WX, Lv XB, Tang QL, Sun LJ, Liu BD (2015). miR-483-5p determines mitochondrial fission and cisplatin sensitivity in tongue squamous cell carcinoma by targeting FIS1. Cancer Lett.

[44] Fan S, Liu B, Sun L, Lv XB, Lin Z, Chen W (2015). Mitochondrial fission determines cisplatin sensitivity in tongue squamous cell carcinoma through the BRCA1-miR-593-5p-MFF axis. Oncotarget.

[45] Wang Y, Huang JW, Calses P, Kemp CJ, Taniguchi T (2012). MiR-96 downregulates REV1 and RAD51 to promote cellular sensitivity to cisplatin and PARP inhibition. Cancer Res.

[46] Xiang Y, Ma N, Wang D, Zhang Y, Zhou J, Wu G (2014). MiR-152 and miR-185 co-contribute to ovarian cancer cells cisplatin sensitivity by targeting DNMT1 directly: a novel epigenetic therapy independent of decitabine. Oncogene.

[47] Yu PN, Yan MD, Lai HC, Huang RL, Chou YC, Lin WC (2014). Downregulation of miR-29 contributes to cisplatin resistance of ovarian cancer cells. Int J Cancer.

[48] Lai X, Gupta SK, Schmitz U, Marquardt S, Knoll S, Spitschak A (2018). MiR-205-5p and miR-342-3p cooperate in the repression of the E2F1 transcription factor in the context of anticancer chemotherapy resistance. Theranostics.

[49] Zhang Q, Zhuang J, Deng Y, Yang L, Cao W, Chen W (2017). miR34a/GOLPH3 Axis abrogates Urothelial Bladder Cancer Chemoresistance via Reduced Cancer Stemness. Theranostics.

[50] Lan CH, Sheng JQ, Fang DC, Meng QZ, Fan LL, Huang ZR (2010). Involvement of VDAC1 and Bcl-2 family of proteins in VacA-induced cytochrome c release and apoptosis of gastric epithelial carcinoma cells. J Dig Dis.

[51] Shang D, Wu J, Guo L, Xu Y, Liu L, Lu J (2017). Metformin increases sensitivity of osteosarcoma stem cells to cisplatin by inhibiting expression of PKM2. Int J Oncol.

[52] Fukuda S, Miyata H, Miyazaki Y, Makino T, Takahashi T, Kurokawa Y (2015). Pyruvate Kinase M2 Modulates Esophageal Squamous Cell Carcinoma Chemotherapy Response by Regulating the Pentose Phosphate Pathway. Ann Surg Oncol.

[53] Zhang XY, Zhang M, Cong Q, Zhang MX, Zhang MY, Lu YY (2018). Hexokinase 2 confers resistance to cisplatin in ovarian cancer cells by enhancing cisplatin-induced autophagy. Int J Biochem Cell Biol.

[54] Zhao Y, Butler EB, Tan M (2013). Targeting cellular metabolism to improve cancer therapeutics. Cell Death Dis.

[55] Roh JL, Park JY, Kim EH, Jang HJ, Kwon M (2016). Activation of mitochondrial oxidation by PDK2 inhibition reverses cisplatin resistance in head and neck cancer. Cancer Lett.

[56] Mercer TR, Neph S, Dinger ME, Crawford J, Smith MA, Shearwood AM (2011). The human mitochondrial transcriptome. Cell.

[57] Ishikawa K, Takenaga K, Akimoto M, Koshikawa N, Yamaguchi A, Imanishi H (2008). ROS-generating mitochondrial DNA mutations can regulate tumor cell metastasis. Science.

[58] Allegra E, Garozzo A, Lombardo N, De Clemente M, Carey TE (2006). Mutations and polymorphisms in mitochondrial DNA in head and neck cancer cell lines. Acta Otorhinolaryngol Ital.

[59] Gerweck LE, Seetharaman K (1996). Cellular pH gradient in tumor versus normal tissue: potential exploitation for the treatment of cancer. Cancer Res.

[60] Denker SP, Huang DC, Orlowski J, Furthmayr H, Barber DL (2000). Direct binding of the Na-H exchanger NHE1 to ERM proteins regulates the cortical cytoskeleton and cell shape independently of H(+) translocation. Mol Cell.

[61] Chen M, Huang SL, Zhang XQ, Zhang B, Zhu H, Yang VW (2012). Reversal effects of pantoprazole on multidrug resistance in human gastric adenocarcinoma cells by down-regulating the V-ATPases/mTOR/HIF- 1alpha/P-gp and MRP1 signaling pathway in vitro and in vivo. J Cell Biochem.

