# Supplementary material for: Decreased expression of mitochondrial miR-5787 contributes to chemoresistance by reprogramming glucose metabolism and inhibiting MT-CO3 translation
Source: Theranostics. 2019 Aug 12;9(20):5739–54. doi: 10.7150/thno.37556 (PMC6735381; doi:10.7150/thno.37556)
Supplement: Supplementary file 1 — Supplementary figures and tables. [file thnov09p5739s1.pdf]

Supplementary data

Supplementary Materials and Methods

Supplementary Figure S1

Supplementary Figure S2

Supplementary Figure S3

Supplementary Figure S4

Supplementary Figure S5

Supplementary Figure S6

Supplementary Table S1

Supplementary Table S2

Supplementary Table S3

Supplementary Table S4

Supplementary Materials and Methods

Isolation of mitochondrial and cytosolic fractions

Subcellular fractions were prepared as described previously [1] . In brief, the cells were washed twice with cold phosphate-buffered saline (PBS), and the pellet was suspended in 0.2 mL of buffer (20 mM 4-(2-hydroxyethyl)-1-piperazineethanesulfonic acid (HEPES), pH 7.5, 10 mM KCl, 1.5 mM $MgCl_2$, 1 mM ethylene glycol-bis(β-aminoethyl ether)-N,N,N',N'-tetraacetic acid (EGTA), 1 mM ethylenediaminetetraacetic acid (EDTA), 1 mM dithiothreitol (DTT), 0.1 mM phenylmethylsulfonyl fluoride (PMSF), and 250 mM sucrose) containing a protease inhibitor cocktail. The cells were homogenized with 12 strokes in a Dounce homogenizer, followed by centrifugation twice at 750 g for 5 min at 4°C to collect nuclei and debris. The mitochondria-enriched heavy membrane pellet was collected by further centrifugation at 10,000 g for 15 min at 4°C, and the supernatants were collected for the cytosolic fractions.

To exclude the contamination of cytoplasmic RNA, isolated mitochondria were subjected to RNase T1 (3000 U/mL) plus Micrococcal nuclease (1000 U/mL) digestion on ice for 30 min in the presence or absence of Triton X-100. After the reaction, mitochondria were pelleted by centrifugation, and RNA was extracted from the pellets with Trizol for qRT-PCR analysis.

miRNA microarray analysis

The miRNA microarrays were performed by Shanghai Biotechnology

Corporation (Shanghai, China) with Agilent human miRNA (8*60K) V19.0. Briefly, cytosol RNA, mitochondrial RNA, and total RNA were extracted and checked for a RIN number to inspect RNA integration by an Agilent Bioanalyzer 2100 (Agilent Technologies, Santa Clara, CA, US). miRNA in different RNAs was labeled by miRNA Complete Labeling and Hyb Kit (Cat#5190-0456, Agilent Technologies, Santa Clara, CA, US) following the manufacturer's instructions in the labeling section. Each slide was then hybridized with 100ng Cy3-labeled RNA using miRNA Complete Labeling and Hyb Kit (Cat#5190-0456, Agilent Technologies, Santa Clara, CA, US) in the hybridization Oven (Cat#G2545A, Agilent Technologies, Santa Clara, CA, US) at 55°C，20rpm for 20 hours according to the manufacturer's instructions in the hybridization section. After hybridization, slides were washed in staining dishes (Cat#121, Thermo Shandon, Waltham, MA, US) with Gene Expression Wash Buffer Kit (Cat#5188-5327, Agilent Technologies, Santa Clara, CA, US). Slides were scanned by Agilent Microarray Scanner (Cat#G2565BA，Agilent Technologies, Santa Clara, CA, US) and Feature Extraction software 10.7 (Agilent Technologies, Santa Clara, CA, US) with default settings. Raw data were normalized by Quantile algorithm, Gene Spring Software 11.0 (Agilent Technologies, Santa Clara, CA, US). The GEO accession number of this miRNA microarray is **GSE115118.**

Protein, RNA, and DNA extraction

The cells were rinsed three times with PBS for 5 min each, followed by the

addition of radioimmunoprecipitation assay (RIPA) buffer (Sigma, Cat# R0278) containing a protease inhibitor cocktail. The cells were lysed for 0.5 h at 4°C, the samples were scraped, and centrifuged at a speed of 12000 rpm for 20 min at 4°C. The supernatants were collected as the total protein. Mitochondrial and cytosolic proteins were extracted from the respective fractions. For RNA extraction, total RNA was extracted by the TRIzol method; pelleted mitochondria, nuclear fractions, and cytosolic fractions were subjected to RNA isolation by the TRIzol and TRIzol LS (Life Technologies, Carlsbad, USA) extraction methods, according to the instructions provided by the manufacturer. The mitochondrial fractions were subjected to DNA extraction with the DNA Extract All Reagents Kit (Applied Biosystems™, 4403319).

qRT-PCR

qRT-PCR was performed using SYBR Green Real-time PCR Master Mix (ReverTra Ace, Toyobo) and a LightCycler 480 (Roche, Basel, Switzerland) according to the manufacturer's instructions. Reactions were performed in triplicate in three independent experiments. The sequences of all primers are listed in Supplementary Table 1. The primers for U6, 6 mitomiRs, premiR-5787 and the negative control miRNA mimics were purchased from RiboBio (Guangzhou, China). The relative expression levels in the control were set to 1.

Western blotting

Immunoblotting was performed as previously described [2]. Protein extracts

were resolved on 8% or 12% SDS-polyacrylamide gel electrophoresis, transferred to polyvinylidene difluoride membranes (BioRad, Berkeley, CA, USA), probed with antibodies against human VDAC1 (Proteintech, 10866-1-AP), TOMM20 (Millipore, MABT166), HK2 (Sangon, AB21853b), PKM2 (Proteintech, 15822-1-AP), MT-CO3 (Abcam, ab110259), DICER (Santa Cruz, sc-30226), AGO2 (Abcam, ab57113), TRBP (Abcam, ab180947), GW182 (Santa Cruz, sc-56314), β-actin (Proteintech, 60008-1-Ig), GAPDH (Proteintech, 60004-1-Ig), MT-CYB (Abcam, ab182330), PACS1 (ABclonal, A12659), and NDST1 (Abcam, ab129248) followed by a peroxidase-conjugated secondary antibody (Proteintech); the bands were visualized by chemiluminescence (GE, Fairfield, CT, USA).

Northern blotting

Northern blotting for miR-5787 was performed as previously reported [3, 4]. Briefly, 10 μg of RNA was fractionated on a 1% agarose gel at 50 V for 1.5 h. The RNA was transferred to a nitrocellulose membrane (Millipore, HATE00010) overnight for at least 16 h. After a wash with 2× saline sodium citrate (SSC) buffer, the nitrocellulose membrane was UV cross-linked at 1,000 μF and prehybridized for 30 min at 52°C in UltraHyb buffer (Roche, 11796895001). The miR-5787 probe was 5’ digoxigenin (DIG) labeled (5’-GGGCTGGGGCGCGGGGAGGT-3’) as described previously [4], and the blot was hybridized in 10 mL of UltraHyb buffer. After 2 washes (5 min) at hybridization temperature in low-stringency buffer, 2 washes (15 min) at hybridization temperature in high-stringency buffer, an incubation (30 min) in

blocking solution, and an incubation (30 min) in Anti-Digoxigenin-AP solution, the blot was analyzed on a phosphorimager (Molecular Dynamics) with CSPD detection buffer. The process was repeated using radiolabeled probes for U6 snRNA (5'-GCAGGGGCCATGCTAATCTTCTCTGTATCG-3')[5] and 12S rRNA (5'-GATGCTTGCATGTGTAATCTTACTA-3') [6].

Transfections

Six miRNA mimics, miR-5787 3' mutant mimics, miR-5787 5' mutant mimics, and negative controls were obtained from RiboBio (Guangzhou, China). The negative control had been confirmed to lack homology with the human mitochondrial genome. PACS1, NDST1，MT-CO3 and GW182 siRNAs were purchased from GenePharma (Shanghai, China). The cells were transfected using the Lipofectamine RNAiMAX Transfection Reagent (Invitrogen™).

ROS production assay

The intracellular ROS levels were measured by detecting the conversion of cell-permeable 2,7-dichlorofluorescein diacetate (DCFH-DA) to fluorescent dichlorofluorescein (DCF) (Beyotime Biotechnology, S0033). The cells were seeded in 6-well plates and treated under different conditions. Subsequently, all wells were washed with PBS three times, and the cells were incubated with DCFH-DA at 37°C for 25 min. The cells were washed with serum-free culture medium. Finally, the DCF fluorescence distribution was detected by a fluorescence microplate reader at an

excitation wavelength of 488 nm and an emission wavelength of 525 nm.

Fluorescence in situ hybridization

Cells were washed with PBS and treated with 0.1 μM MitoTracker Red CMXRos (Molecular Probes) for 30 min to label the mitochondria. Next, the cells were washed with PBS and fixed with 4% formaldehyde in PBS (pH 7.4) for 20 min at room temperature. The cells were then permeabilized in PBS containing 0.1% Triton X-100 at room temperature for 15 min, washed with PBS for 3×5 min, digested with 0.05% trypsin, and subjected to a series of dehydrations with 75%, 85%, and 100% ethanol prior to hybridization. Using a DIG-labeled miR-5787 probe, hybridization was performed in a hybridization solution (probe dilution 1:1000) for 16 h at 52°C in a moist chamber. Subsequently, the cells were washed for 30 min in 2×SSC at 52°C and then for 30 min in 25% deionized formamide/2×SSC at 52°C. For confocal microscopy, the cells on coverslips were counterstained with DAPI and imaged using a confocal microscope TCS SP5 (Lecia, Solms, Germany). Image J software (NIH, Bethesda, MD) was used to analyze the colocalization of miR-5787 and mitochondria.

Plasmid construction and establishment of stable cell lines

Lentiviral plasmids (pLVX-mCMV-tdTomato-PGKPuro) delivering miR-5787 precursors were purchased from Biowit Technologies (Shenzhen, China). miR-5787 sponge and negative controls were purchased from GenePharma (Shanghai, China).

The sponge was designed using 4 repeated reverse complementary sequences of miR-5787 and cloned into the lentiviral vector LV3(H1/GFP&Puro). The miR-5787 sponge sequence was GATCCACCTCCCCGCGCCCCAGCCCCGATACCTCCCCGCGCCCCAGCCCTCACACCTCCCCGCGCCCCAGCCCTTTTTTGAATT and its negative control sequence was GATCC TTCTCCGAACGTGTCACGTCGATTTCTCCGAACGTGTCACGTACCGGTTTCTCCGAACGTGTCACGTTCACTTCTCCGAACGTGTCACGTTTTTTGAATT. The viruses were amplified in HEK293 cells. Lentiviral infection and the construction of the Cal27, Scc25, Cal27-re and Scc25-re stable cell lines were performed as previously described.

CCK-8 assay

After various treatments, the cells were collected and 10000 cells/well were plated into 96-well plates and incubated overnight. The number of cells was determined using a cell counting kit-8 (CCK-8, Dojindo, Kumamoto, Japan) at 0, 24, 48, 72, and 96 h. The medium of each well was removed, and a mixture of 10 ul of CCK-8 and 90 ul of DMEM or DMEM/F12 was added. The plates were incubated for an additional 2.5 h, and the absorbance at 450 nm was measured using a microplate reader (Multiskan MK3, Shanghai, China).

Modified Boyden chamber assay

1×10^5 cells were plated into the upper chamber of a polycarbonate Transwell filter chamber (Corning, NewYork, NY, USA) and incubated for 22 h. For invasion assay, the upper chamber was coated with Basement Membrane (R&D, Minneapolis, MN, USA). Cells on the lower membrane surface were fixed in 4% paraformaldehyde, stained with crystal violet and counted (5 random 100× fields per well). Three independent experiments were performed, and the data are presented as the average ± S.D.

Glucose metabolism PCR array

The glucose metabolism PCR array was performed by using a series of kits from Yingbiotech (Shanghai, China) (Cat. No. PAHS-006Z). Briefly, total RNA of Cal27 and Cal27-re was extracted with an RNeasy Mini Kit (Qiagen, 4104). RNA yield and quality were assessed with UV absorbance and denaturing agarose gel electrophoresis. cDNA synthesis was performed with the RT2 First Strand Kit (Qiagen, 330401). Real-time PCR was performed with the RT2 SYBR Green Mastermix (Qiagen, 330401). The primers for glycolysis genes and oxidative phosphorylation genes were applied in the real-time PCR. Glycolysis genes included ALDOA, ALDOB, ALDOC, BPGM, ENO1, ENO2, ENO3, GALM, GCK, GPI, HK2, HK3, PFKL, PGAM2, PGK1, PGK2, PGM1, PGM2, PGM3, PKLR, and TPI1. Oxidative phosphorylation genes included ACLY, ACO1, ACO2, CS, DLAT, DLD, DLST, FH, IDH1, IDH2, IDH3A, IDH3B, IDH3G, MDH1, MDH1B, MDH2, OGDH, PC, PCK1, PCK2, PDHA1, PDHB, SDHA, SDHB, SDHC, SDHD, SUCLA2,

SUCLG1, and SUCLG2. Reactions were performed in triplicate in three independent experiments.

Immunofluorescence staining

Prior to immunofluorescence staining, MitoTracker Red CMXRos was used to label mitochondria. Cal27 and Cal27-re cells were incubated with primary antibodies against AGO2 (Abcam, ab57113) and then incubated with rhodamine- or FITC-conjugated secondary antibodies (Invitrogen). The coverslips were counterstained with 46-diamidino-2-phenyl indole and imaged under a confocal microscope TCS SP5 (Lecia, Solms, Germany).

Dual luciferase assays

Plasmids for luciferase reporter assays were constructed by inserting target sequences into the psiCheck2 vector (Promega). The reporter was transfected into cells that had been previously treated with control RNA or miRNAs for 12 h. Dual luciferase assay was performed after plasmid transfection for 48 h using the luciferase assay kit from Promega. The standard derivations were based on triplicate experiments.

RNA immunoprecipitation

RNA immunoprecipitation was performed using the Magna RIP RNA-Binding Protein Immunoprecipitation Kit (17-700, Millipore, Billerica, MA) according to the

manufacturer's instructions. Briefly, mitochondria were collected form Cal27 and Cal27-re cells, washed twice with PBS at room temperature followed by incubation with immunoprecipitation (IP) lysis buffer for 20 min at 4°C. The samples were removed from the dish and centrifuged for 15 min at 12000 g and 4°C. The supernatant was divided into two parts, anti-AGO2 antibody (Abcam, ab57113) and IgG were added, followed by rotation at 20 rpm for 1 h. Protein beads were added to each tube, followed by rotation at 20 rpm for 0.5 h. A magnetic frame was applied to remove the supernatant, followed by three washes with lysis buffer. Protease K and RNase inhibitor were added to the lysis buffer followed by rotation for 30 min at 55°C to remove protein. TRIZOL LS was added to the remaining sample, and RNA was then extracted. qRT-PCR was applied to assess miR-5787, miR-5100, MT-CO3 and MT-ND5 expression in the immunocomplex.

Tumor xenografts

Male BALB/c-nu mice were chosen to evaluate the effects of miR-5787 on TSCCs in vivo. Cal27 cells with stable knockdown of miR-5787 were used, with Cal27 and Cal27+miR sponge-NC as controls. Cal27-re cells with stably upregulated miR-5787 were used, with Cal27-re and Cal27-re+miR-NC as controls. Six mice 4 to 6 weeks of age were used in each group. 5×10^6 cells in 150 μL of PBS were injected subcutaneously into the flanks of the nude mice. One week after implantation, when the tumor became palpable with a diameter of ~2 mm, cisplatin (5 mg/kg) was administered via intraperitoneal injections every three days from days 8 to 32. The

tumor size was measured and calculated on the day of cisplatin injection according to the following formula: TV (mm^3) =length×width^2×0.5. On day 35, the primary tumors were carefully removed, imaged, and analyzed by IHC, ISH, Western blots and qRT-PCR.

In situ hybridization

In situ hybridization (ISH) was performed as previously described [2], according to the manufacturer's protocol (Exiqon, Vedbaek, Denmark). Briefly, after demasking, miR-5787 was hybridized to 5' DIG-labeled probe (Boster Biological Technology), which was recognized by a specific anti-DIG antibody that was directly conjugated to alkaline phosphatase. The nuclei were counterstained with Nuclear Fast Red Solution (N3020, Sigma) and 5 × 200 tumor cells were counted randomly in each section. Sections with more than 300 miR-5787-positive cells were considered to have high miR-5787 expression.

Immunohistochemistry

TSCC sections were incubated with an MT-CO3 antibody (Abcam, ab116897) at 37°C for 1 h. The sections were then treated with a secondary antibody followed by further incubation with a streptavidin-horseradish peroxidase complex. Diaminobenzidine (Dako, Carpinteria, CA, USA) was used as a chromogen, and the nuclei were counterstained with hematoxylin. Then, 5 × 200 tumor cells were counted in each section. Sections with more than 350 MT-CO3-positive cells were considered to have high MT-CO3 expression.

References

1. Fan S, Chen WX, Lv XB, Tang QL, Sun LJ, Liu BD, et al. miR-483-5p determines mitochondrial fission and cisplatin sensitivity in tongue squamous cell carcinoma by targeting FIS1. Cancer Lett. 2015; 362: 183-91.
2. Sun L, Yao Y, Liu B, Lin Z, Lin L, Yang M, et al. MiR-200b and miR-15b regulate chemotherapy-induced epithelial-mesenchymal transition in human tongue cancer cells by targeting BMI1. Oncogene. 2012; 31: 432-45.
3. Yu F, Yao H, Zhu P, Zhang X, Pan Q, Gong C, et al. let-7 regulates self renewal and tumorigenicity of breast cancer cells. Cell. 2007; 131: 1109-23.
4. Takamizawa J, Konishi H, Yanagisawa K, Tomida S, Osada H, Endoh H, et al. Reduced expression of the let-7 microRNAs in human lung cancers in association with shortened postoperative survival. Cancer Res. 2004; 64: 3753-6.
5. Cimmino A, Calin GA, Fabbri M, Iorio MV, Ferracin M, Shimizu M, et al. miR-15 and miR-16 induce apoptosis by targeting BCL2. Proc Natl Acad Sci U S A. 2005; 102: 13944-9.
6. Wu H, Sun H, Liang X, Lima WF, Crooke ST. Human RNase H1 is associated with protein P32 and is involved in mitochondrial pre-rRNA processing. Plos One. 2013; 8: e71006.

Supplementary figures

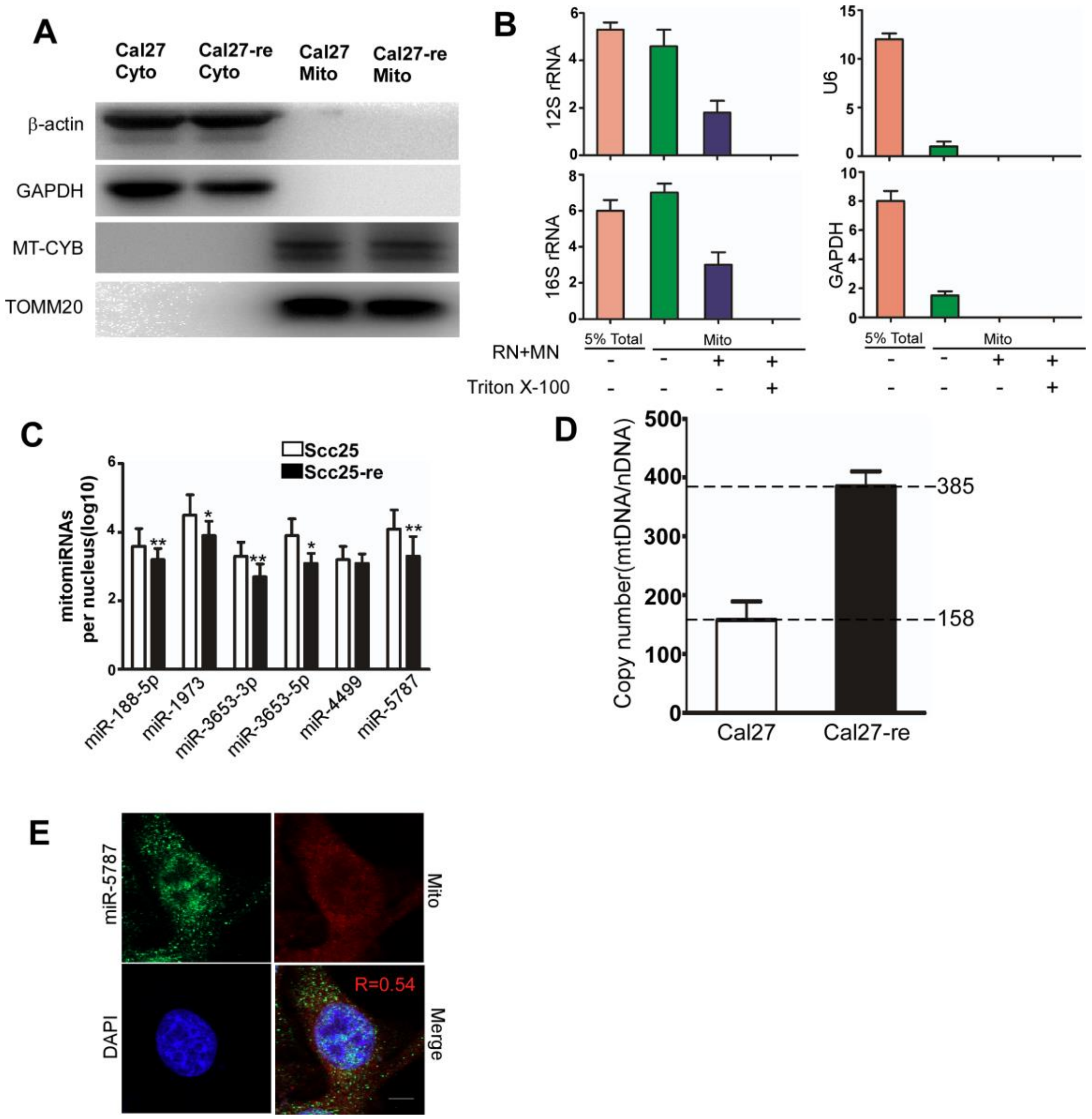

Supplementary Figure S1. Purity of isolated mitochondria and miRNA profiles in Cal27 and Cal27-re cells. **(A)** Quantification of mitochondrial protein purity by Western blots showing that two cytosolic proteins, β-actin and GAPDH, were almost negative in the mitochondrial preparations of Cal27 and Cal27-re cells. Additionally, the cytosolic fraction barely contained any mitochondrial proteins, such as MT-CYB or TOMM20. **(B)** Degradation of cytoplasmic RNA in the isolated mitochondria of Cal27 cells. Three aliquots of purified mitochondria were treated, one with isolation buffer,

one with RNase T1 (RN T1) plus micrococcal nuclease (MNase), and one with the combination of nucleases in the presence of Triton X-100. **(C)** Quantification of the number of copies of six mitomiRs per Scc25 and Scc25-re cell nucleus. Five mitomiRs were significantly upregulated in Scc25 cells. **P*<0.01, ***P*<0.001. **(D)** Mitochondrial copy number per nucleus in Cal27 and Cal27-re cells. **(E)** Fluorescence in situ hybridization indicated that miR-5787 co-localize with the mitochondria in Cal27-re cells. Co-localization coefficient (R) equals 0.54. The scale bar equals 3 μm.

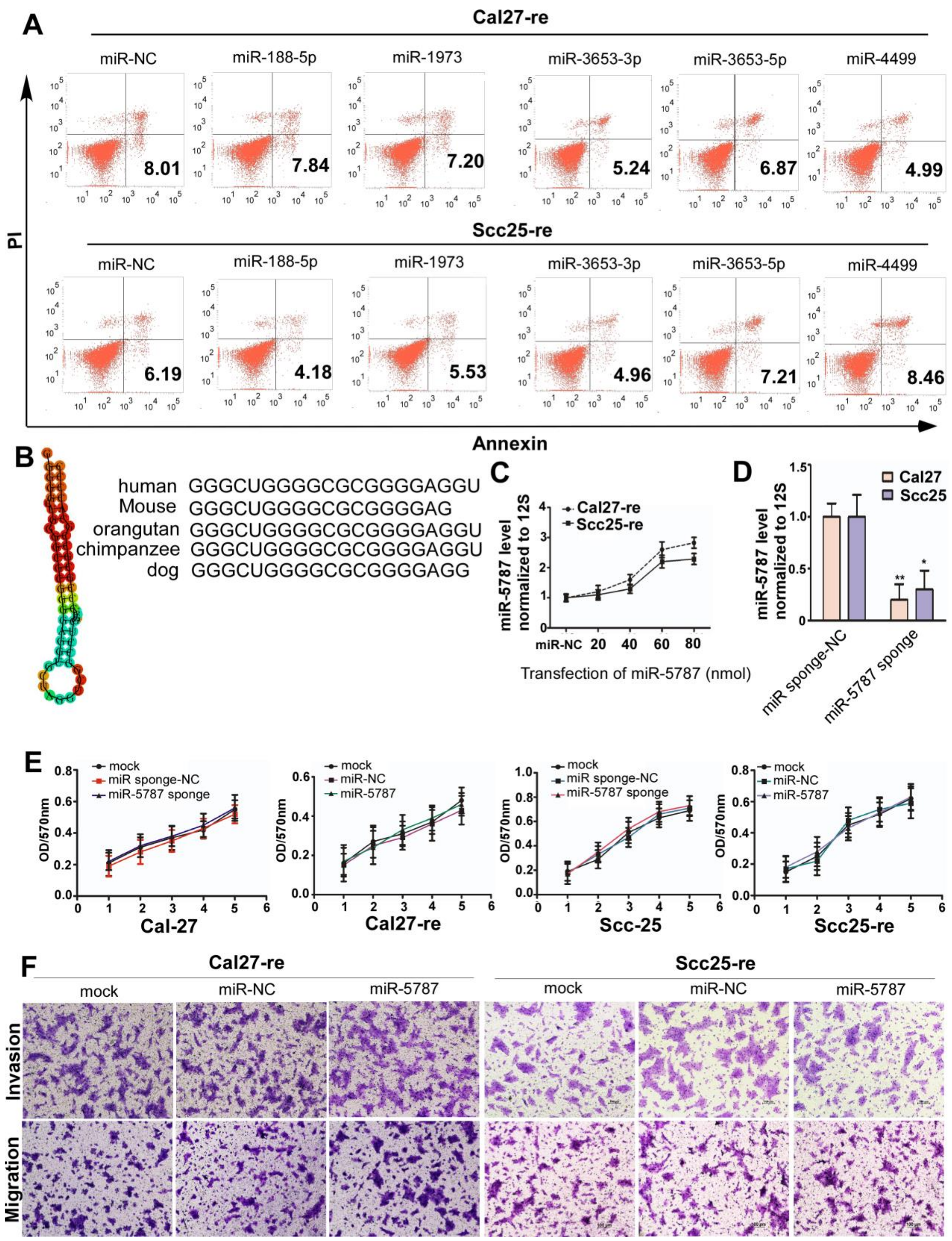

Supplementary Figure S2 (A) Flow cytometry shows the effect of miR-188-5p, miR-1973, miR-3653-3p, miR-3653-5p and miR-4499 on the apoptosis of TSCC cells induced by cisplatin. **(B)** Predicted secondary structure of the premiR-5787 using the computer program RNAfold. The conservation of miR-5787 in vertebrate species. The conservation of miR-5787 is 100% in orangutans and chimpanzees. **(C)** A

transfection concentration investigation indicated that 60 nmol was a suitable concentration for upregulating miR-5787 in Cal27-re and Scc25-re cells. **(D)** miR-5787 sponge could efficiently downregulate miR-5787 in Cal27 and Scc25 cells. **P*<0.01, ***P*<0.001. **(E)** Proliferation of TSCC cells were detected using a CCK-8 assay **(F)** Effects of miR-5787 on the invasion and migration of TSCC cells.

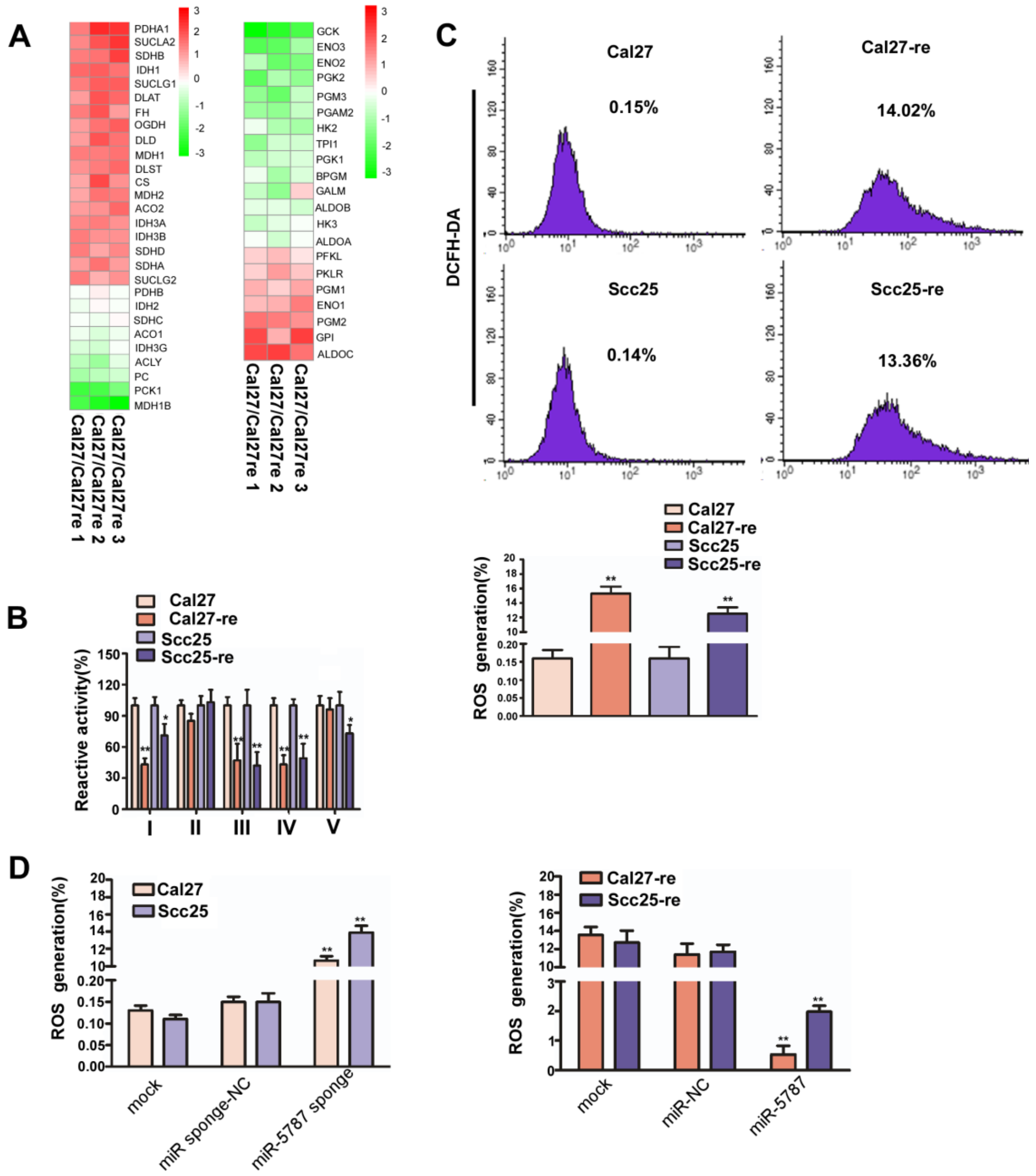

Supplementary Figure S3 (A) Heat map showing the metabolic changes in Cal27 and Cal27-re cells, with the left and right panels representing the OXPHOS glycolysis

changes, respectively **(B)** Functional changes in mitochondrial respiratory chain complexes I-V in Cal27/Cal27-re cells and Scc25/ Scc25-re cells. The results show that complexes I, III, and IV were decreased in Cal27-re cells, and complexes I, III, IV, and V were decreased in Scc25-re cells compared to the levels in the parental cell lines. **P*<0.01, ***P*<0.001. **(C)** ROS assay showing that Cal27-re and Scc25-re cells generated more ROS than their parental cell lines. ***P*<0.001. **(D)** miR-5787 regulated ROS generation in two pairs of TSCC cells. ***P*<0.001.

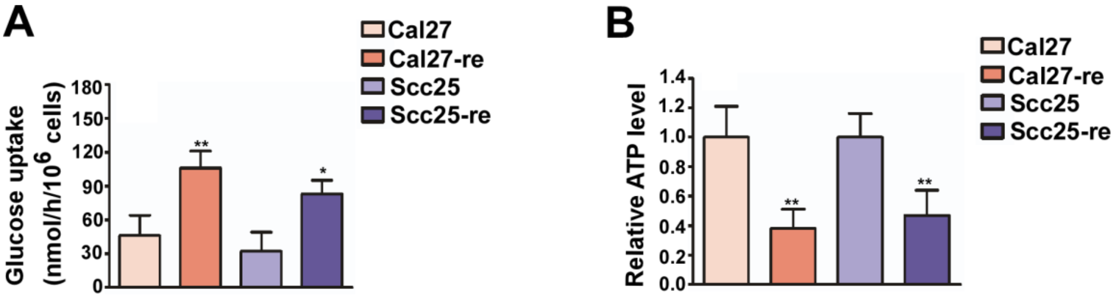

Supplementary Figure S4 (A) The glucose uptake in Cal27/Cal27-re cells and Scc25 /Scc25-re cells. The results show that the uptake of glucose in the chemoresistant cells was more than their parental cells. **P*<0.01, ***P*<0.001. **(B)** The ATP synthesis in Cal27/Cal27-re cells and Scc25/Scc25-re cells. The results showed that chemoresistant cells produced less ATP than their parental cells. ***P*<0.001.

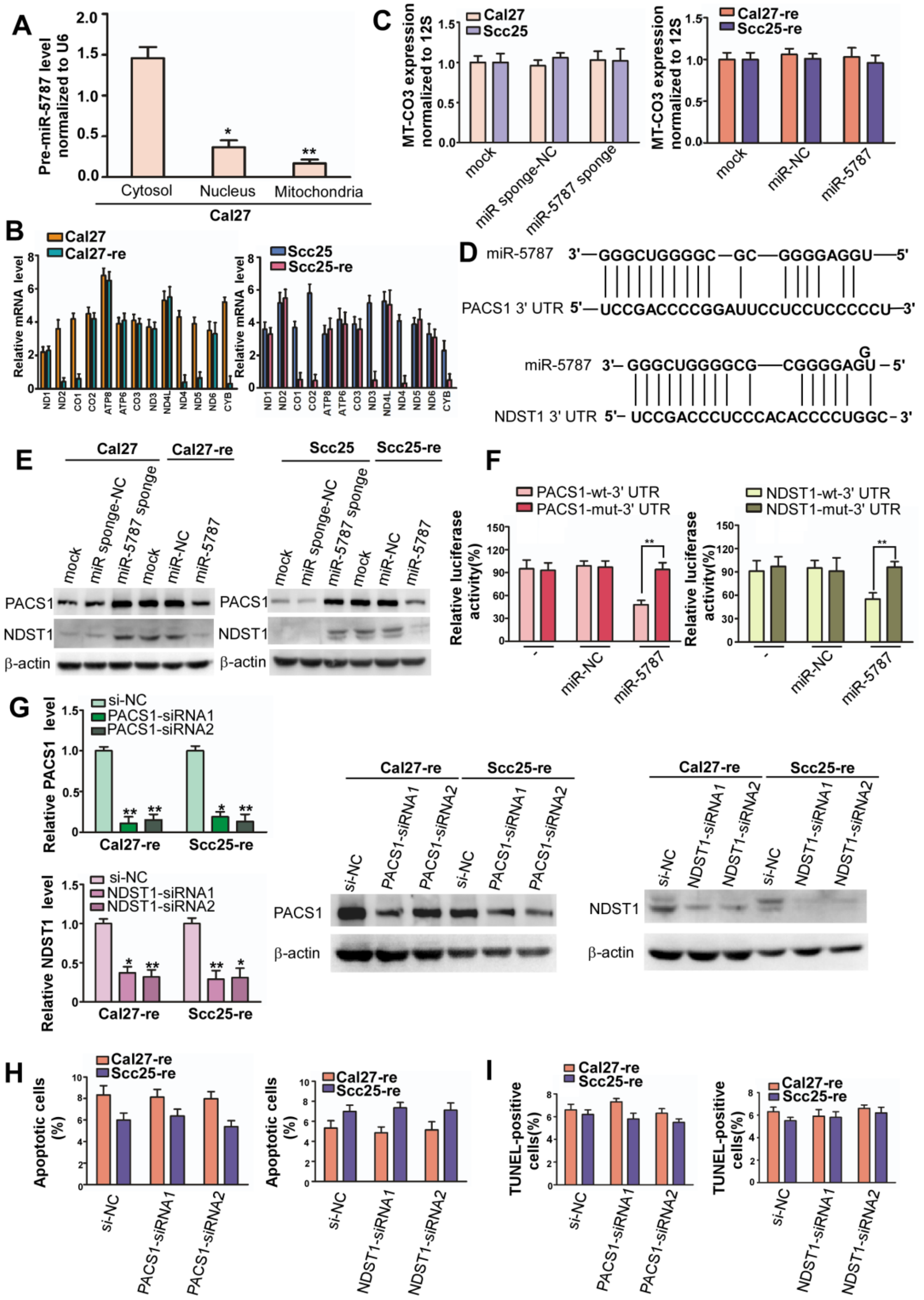

Supplementary Figure S5 (A) qRT-PCR showing the amount of pre-miRNA-5787 in the total cell fraction versus other fractions. **P*<0.01 versus cytosol fraction and ***P*<0.001 versus nucleus. **(B)** qRT-PCR showing the expression of 13 mitochondrial

genes in Cal27/Cal27-re and Scc25/Scc25-re cells. **(C)** qRT-PCR indicates that MT-CO3 RNA level had no change with the treatment of miR-5787 mimics or sponge. **(D)** Target prediction showing that miR-5787 might target the 3'UTRs of PACS1 and NDST1. **(E)** Western blots confirming that miR-5787 could downregulate the protein levels of PACS1 and NDST1. β-actin was used as an internal control. **(F)** Luciferase assays confirming that miR-5787 could target the wild-type (wt) rather than the mutant (mut) 3'UTRs of PACS1 and NDST1. $^{**}P<0.001$. **(G)** Knockdown of PACS1 and NDST1 in Cal27-re and Scc25-re cells with siRNA, as confirmed by qRT-PCR and Western blots. β-actin was used as an internal control. $^{*}P<0.01$, $^{**}P<0.001$. **(H)** Flow cytometry showing that the knockdown of PACS1 and NDST1 had almost no effect on the apoptosis of TSCC cells. **(I)** TUNEL assay further confirming that the knockdown of PACS1 and NDST1 had no effect on apoptosis in TSCC cells.

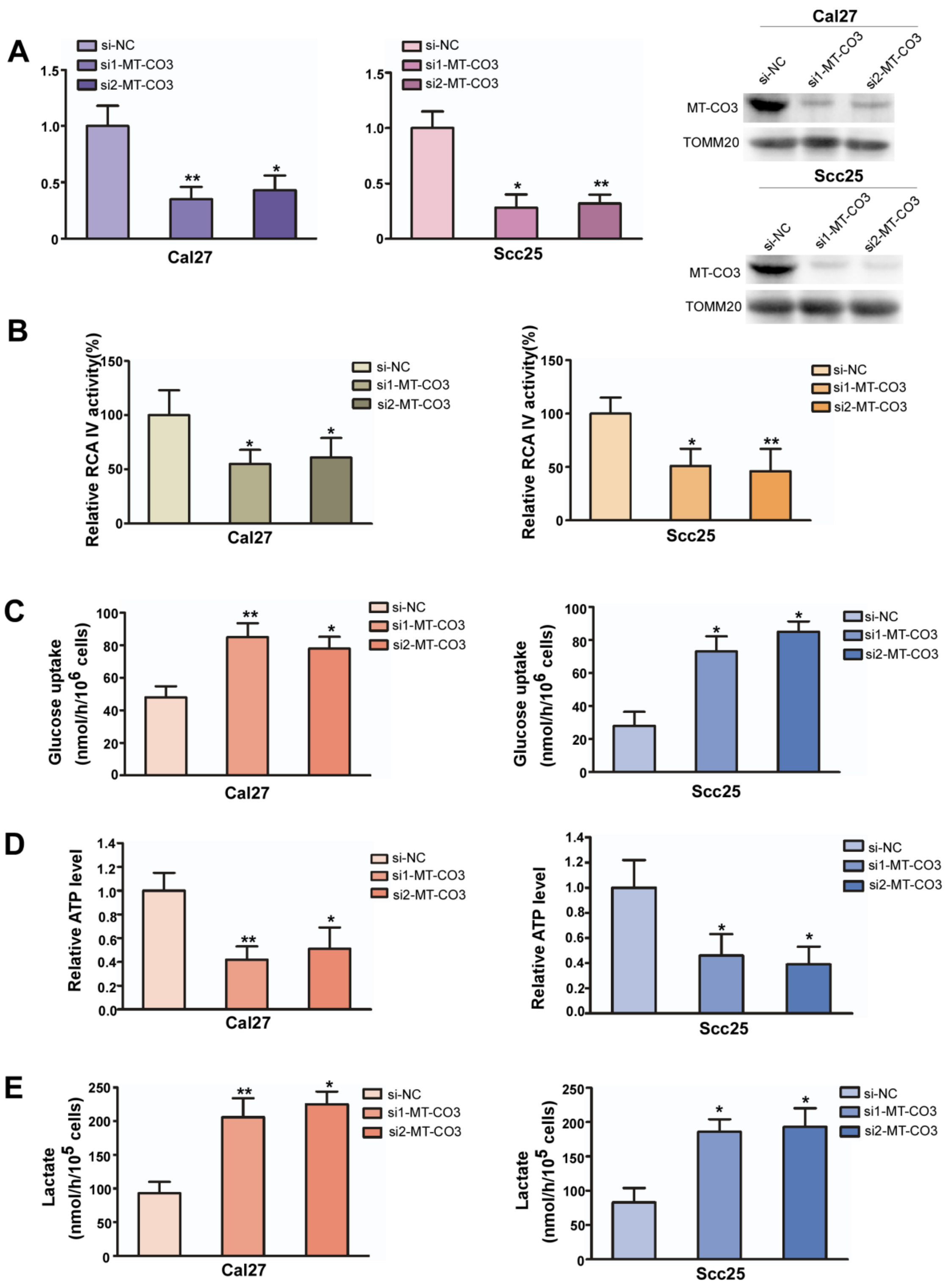

Supplementary Figure S6 (A) Knockdown of MT-CO3 in Cal27 and Scc25 cells with siRNA, as confirmed by qRT-PCR and Western blots. TOMM20 was used as an internal control. **P*<0.01, ***P*<0.001. **(B)** Fold changes in mitochondrial respiratory chain complexes IV in Cal27 and Scc25 cells. **P*<0.01, ***P*<0.001. **(C)** The change of

glucose uptake in Cal27 and Scc25 cells. *P<0.01, **P<0.001. **(D)** The change of ATP synthesis in Cal27 and Scc25 cells. *P<0.01, **P<0.001. **(E)** The change of lactate generation in Cal27 and Scc25 cells. *P<0.01, **P<0.001.

Supplementary Tables

Supplementary Table S1. Primers used in present study

Gene	Direction	Primer sequence
qPCR		
β-actin	Forward	5'-GGGAAATCGTGCGTGACATTAAGG-3'
	Reverse	5'-CAGGAAGGAAGGCTGGAAGAGTG-3'
GAPDH	Forward	5'-CGCTGAGTACGTCGTGGAGTC-3'
	Reverse	5'-GCTGATGATCTTGAGGCTGTTGTC-3'
MT-CYTB	Forward	5'-TAGCGGATGATTCAGCCATAA-3'
	Reverse	5'-ATGACCCCAATACGCAAAACT-3'
MT-ND5	Forward	5'-GAGGGTGATGGTAGATGTGGC-3'
	Reverse	5'-CCTAATGCTTACCGAACGAAA-3'
qRT-PCR		
MT-CYB	Forward	5'-CAAACTAGGAGGCGTCCTTG-3'
	Reverse	5'-CTGGTTGTCCTCCGATTCAG-3'
MT-ND5	Forward	5'-CTATCACCACTCTGTTCGCAG-3'
	Reverse	5'-GTGGTTGGTTGATGCCGATTG-3'
β-actin	Forward	5'-CTACCTCATGAAGATCCTCACCGA-3'
	Reverse	5'-TTCTCCTTAATGTCACGCACGATT-3'
GAPDH	Forward	5'-GGACCTGACCTGCCGTCTAG-3'
	Reverse	5'-GTAGCCCAGGATGCCCTTGA-3'
12S rRNA	Forward	5'-CACTACGAGCCACAGCTTAA-3'
	Reverse	5'-TCAGGGTTTGCTGAAGATGG-3'
5S rRNA	Forward	5'-TTCAGCGTCTACGGCCATAC-3'
	Reverse	5'-AGCCAAAGAAAAAGCCTAC-3'
AGO2	Forward	5'-CCCCGCCAAGAGGGTTAGAGC-3'
	Reverse	5'-AGGTCACGCTGCCAGGAGAAG-3'
MT-CO3	Forward	5'-CACATCCGTATTACTCGCATC-3'
	Reverse	5'-GAAGTACTCTGAGGCTTGTAG-3'
MT-ND2	Forward	5'-CTACGCCTAATCTACTCCAC-3'
	Reverse	5'-CTTTGAAGGCTCTTGGTCTG-3'

MT-ND6	Forward	5'-CTAAAACACTCACCAAGACC-3'
	Reverse	5'-GGAATGATGGTTGTCTTTGG-3'
MT-ND1	Forward	5'-CCTAGGCCTCCTATTTATTC-3'
	Reverse	5'-GAATGATGGCTAGGGTGAC-3'
MT-ND3	Forward	5'-GCGGCTTCGACCCTATATCC-3'
	Reverse	5'-GTTAGTTGTTTGTAGGGCTCATGGT-3'
MT-ND4	Forward	5'-GGACTCCACTTATGACTCCC-3'
	Reverse	5'-GGTTGAGAATGAGTGTGAGGC-3'
MT-ND4L	Forward	5'-TCGCTGTTCATTATAGCTACTCTCATAA-3'
	Reverse	5'-CGCAGGCGGCAAAGACTA-3'
MT-ND5	Forward	5'-CTATCACCACTCTGTTCGCAG-3'
	Reverse	5'-GTGGTTGGTTGATGCCGATTG-3'
MT-CYTB	Forward	5'-CAAACTAGGAGGCGTCCTTG-3'
	Reverse	5'-CTGGTTGTCCTCCGATTCAG-3'
MT-COX1	Forward	5'-GATTTTTCGGTCACCCTGAAG-3'
	Reverse	5'-CTCAGACCATACCTATGTATC-3'
MT-COX2	Forward	5'-CTATCCTGCCCGCCATCATC-3'
	Reverse	5'-GATTAGTCCGCCGTAGTCGG-3'
MT-ATP6	Forward	5'-TCGGTTGTTGATGAGATATTTGGA-3'
	Reverse	5'-CGCCGCAGTACTGATCATTCT-3'
MT-ATP8	Forward	5'-CCCTCACCAAAGCCCATAAA-3'
	Reverse	5'-GAATGAAGCGAACAGATTTTCGT-3'
16S rRNA	Forward	5'-ACCGGAGTAATCCAGGTCGG-3'
	Reverse	5'-AGGCGCTTTGTGAAGTAGGC-3'x

Supplementary Table S2. Quantification of the mitochondrial to nuclear DNA ratio in the mitochondrial fraction using two mitochondrial (MT-CYB and MT-ND5) and two nuclear genes (GAPDH and ACTB). GAPDH was used to normalize the results, and ACTB was used as a calibrator to calculate the DNA ratio. The results show strong enrichment in mitochondria and the absence of nuclear DNA contamination in the mitochondrial fraction.

Gene	Description	CP	Mitochondrial to nuclear DNA ratio(2-DDCT)
MT-CYB	mitochondrial encoded cytochrome b	17.35	21321
MT-ND5	mitochondrial encoded NADH dehydrogenase 5	17.85	15076
GAPDH	Nuclear gene GAPDH taken as calibrator	31.73	1
ACTB	Nuclear gene ACTB taken as endogenous control	33.17	0

Supplementary Table S3. Relative expression of two nuclear genes (ACTB and GAPDH) and two mitochondrial genes (MT-CYB and MT-ND5) in the mitochondrial RNA extract relative to the cytosolic RNA extract. The results show the low contamination level of the mitochondrial fraction by genomic mRNA.

Control gene	ACTB	GAPDH	MT-CYB	MT-ND5
Mean CP in cytoplasmic extract	12	15.28	32.68	35.45
Mean CP in mitochondrial extract	31.22	36.35	23.57	27.58
Relative expression in mitochondrial/cytosolic RNA extract(fold changes)	1.64×10^{-6}	4.54×10^{-7}	553	234

Supplementary Table S4. miRNA target prediction showing that miR-3653-3p might target MT-ND3 or MT-ND5, miR-3653-5p might target MT-CO3, miR-4499 might target MT-ND5, and miR-5787 might target MT-CO3.

<table>
<tr><th>miRNA</th><th>length</th><th>target</th><th>target_length</th><th>start</th><th>hairpin_energy(kcal/mol)</th><th>pvalue</th><th>score</th></tr>
<tr><td>>hsa-miR-3653-3p</td><td>18</td><td>MT-ND3</td><td>346</td><td>164</td><td>-13.1</td><td>1</td><td>2.0616552</td></tr>
<tr><td>miRNA 3’ GAAGUCAGUUGAAGAAUC- 5’</td><td></td><td></td><td></td><td></td><td></td><td></td><td></td></tr>
<tr><td>||||||||</td><td></td><td></td><td></td><td></td><td></td><td></td><td></td></tr>
<tr><td>target 5’ ---------UCUUCUUAGU 3’</td><td></td><td></td><td></td><td></td><td></td><td></td><td></td></tr>
<tr><td>>hsa-miR-3653-3p</td><td>18</td><td>MT-ND5</td><td>1809</td><td>763</td><td>-13.5</td><td>1</td><td>1.1584893</td></tr>
<tr><td>miRNA 3’ GAAGUCAGUU-GAAGAAUC 5’</td><td></td><td></td><td></td><td></td><td></td><td></td><td></td></tr>
<tr><td>||| || |||||||</td><td></td><td></td><td></td><td></td><td></td><td></td><td></td></tr>
<tr><td>target 5’ --GCAGG-AAUCUUCUUAC 3’</td><td></td><td></td><td></td><td></td><td></td><td></td><td></td></tr>
<tr><td>>hsa-miR-3653-5p</td><td>20</td><td>MT-CO3</td><td>784</td><td>415</td><td>-19.8</td><td>0.99429</td><td>0.3064692</td></tr>
<tr><td>miRNA 3’ CUUCUUCUUAGUAGUCCUCC 5’</td><td></td><td></td><td></td><td></td><td></td><td></td><td></td></tr>
<tr><td>|||||||||</td><td></td><td></td><td></td><td></td><td></td><td></td><td></td></tr>
<tr><td>target 5’ ---------GCAUCAGGAGU 3’</td><td></td><td></td><td></td><td></td><td></td><td></td><td></td></tr>
<tr><td>>hsa-miR-4499</td><td>17</td><td>MT-ND5</td><td>1809</td><td>127</td><td>-19</td><td>0.999999</td><td>2.2127611</td></tr>
<tr><td>miRNA 3’ AGGGAGGAG-AGUCAGAA 5’</td><td></td><td></td><td></td><td></td><td></td><td></td><td></td></tr>
<tr><td>||:|: |: |||||||</td><td></td><td></td><td></td><td></td><td></td><td></td><td></td></tr>
<tr><td>target 5’ ACCUUUAUUAUCAGUCUC 3’</td><td></td><td></td><td></td><td></td><td></td><td></td><td></td></tr>
<tr><td>>hsa-miR-5787</td><td>20</td><td>MT-CO3</td><td>784</td><td>71</td><td>-31.7</td><td>0.044499</td><td>0.041771329</td></tr>
<tr><td>miRNA 3’ UGGAGGG--GC-GCGGGGUCGGG- 5’</td><td></td><td></td><td></td><td></td><td></td><td></td><td></td></tr>
<tr><td>|||||: :| | |:||:|||:</td><td></td><td></td><td></td><td></td><td></td><td></td><td></td></tr>
<tr><td>target 5’ CCCUCCUAAUGAC-CUCCGGCCUA 3’</td><td></td><td></td><td></td><td></td><td></td><td></td><td></td></tr>
</table>
